# Genomic analysis of Oceanotoga teriensis strain UFV_LIMV02, a multidrug-resistant thermophilic bacterium isolated from an offshore oil reservoir

**DOI:** 10.1099/acmi.0.000801.v3

**Published:** 2024-08-15

**Authors:** Adriele Jéssica do Carmo Santos, Roberto Sousa Dias, Carlos Henrique Martins da Silva, Pedro Marcus Pereira Vidigal, Maíra Paula de Sousa, Cynthia Canedo da Silva, Sérgio Oliveira de Paula

**Affiliations:** 1 Department of Microbiology, Federal University of Viçosa, Av. Peter Henry Rolfs, s/n, Campus Universitário, 36570-900, Viçosa, Minas Gerais, Brazil; 2 Department of General Biology, Federal University of Viçosa, Av. Peter Henry Rolfs, s/n, Campus Universitário, 36570-900, Viçosa, Minas Gerais, Brazil; 3 Center for Biomolecules Analysis (NuBIOMOL), Federal University of Viçosa, Vila Gianetti, Campus Universitário, 36570-900, Viçosa, Minas Gerais, Brazil; 4 Leopoldo Américo Miguez de Mello Research and Development Center, Petrobras, Av. Horácio Macedo, 950, Federal University of Rio de Janeiro, 21941-915, Rio de Janeiro, Brazil

**Keywords:** antibiotic and oil reservoir, genomic analysis, *Oceanotoga teriensis*

## Abstract

Bacteria of the species *Oceanotoga teriensis* belong to the family *Petrotogaceae*, are Gram-negative bacilli, are moderately thermophilic and are included in the group of thiosulfate-reducing bacteria, being capable of significantly accelerating corrosion in metallic structures. However, no in-depth study on the genome, antibiotic resistance and mobile elements has been carried out so far. In this work, the isolation, phenotypic and genotypic characterization of the multi-resistant *O. teriensis* UFV_LIMV02 strain was carried out, from water samples from an offshore oil extraction platform in Rio de Janeiro (Brazil). We determined that the isolate has a genome of 2 812 778 bp in size, with 26 % GC content, organized into 34 contigs. Genomic annotation using Rapid Annotation using Subsystem Technology revealed the presence of genes related to resistance to antibiotics and heavy metals. By evaluating the antimicrobial resistance of the isolate using the disc diffusion technique, resistance was verified for the classes of antibiotics, beta-lactams, fluoroquinolones, aminoglycosides, sulfonamides, lincosamides and rifamycins, a total of 14 antibiotics. The search for genomic islands, prophages and defence systems against phage infection revealed the presence of five genomic islands in its genome, containing genes related to resistance to heavy metals and antibiotics, most of which are efflux pumps and several transposases. No prophage was found in its genome; however, nine different defence systems against phage infection were detected. When analysing the clustered regularly interspaced short palindromic repeat (CRISPR) systems, four CRISPR arrays, classified as types I-B and III-B, with 272 spacers, can provide the strain with immunity to different mobile genetic elements and bacteriophage infection. The results found in this study show that the isolate UFV_LIVM02 is an environmental bacterium, resistant to different classes of antibiotics, and that the proteins encoded by the predicted genomic islands may be associated with the development of greater resistance to antibiotics and heavy metals. They provide evidence that environmental bacteria found in offshore oil exploration residues may pose a risk for the spread of antibiotic resistance genes. More comprehensive studies on the microbial community present in oil waste are needed to assess the risks of horizontal gene transfer.

**Impact Statement**

The species *Oceanotoga teriensis* has already been identified in different environments, mainly in oil exploration sites. They are microorganisms capable of fermenting numerous carbohydrates into organic acids, which serve as a carbon source for other bacteria, allowing the maintenance of the metabolic network in environments related to oil exploration and processing and consequently accelerating the microbiologically induced corrosion process. Furthermore, *O. teriensis* can reduce thiosulfate and elemental sulphur to hydrogen sulfide (H_2_S), significantly intensifying the steel corrosion process. However, to our knowledge, there are no genomic analyses to identify antimicrobial resistance genes, genomic islands, prophages and phage immunity systems for this microorganism. This study revealed that the isolate UFV_LIVM02 is resistant to fourteen antibiotics, from different classes, and the *in silico* analysis revealed the presence of resistance genes with low similarity with antibiotic resistance genes (ARGs) deposited in databases, being an indication of new ARG yet uncharacterized; in addition, the presence of five genomic islands containing genes related to resistance to heavy metals and antibiotics was verified, the majority of which were efflux pumps and several transposases. No prophage was found in its genome, but nine different defence systems against phage infection were detected. The results found in this study provide evidence that microorganisms found in offshore oil exploration residues may pose a risk for the spread of ARGs and other mobile genetic elements.

## Data Summary

The sequence of this study is available from the National Centre for Biotechnology Information (NCBI) under BioProject accession number PRJNA837603. The raw data generated by the sequence reads generated by Illumina sequencing were deposited in the sequence read archive at the NCBI under accession number SAMN 28208037. The genomic sequence was deposited in GenBank under accession number JAMHJO000000000.1. The programmes used for assembly, analysis of raw sequences and genetic comparison based on whole genome sequencing are available as described in the methods.

## Introduction

The species *Oceanotoga teriensis* is the only representative of the genus *Oceanotoga* [1,2]. The first strain was named OCT74^T^, isolated from production fluid in offshore oil wells at Bombay High (West India) in 2011 [1]. After its initial description in an oil reservoir, *O. teriensis* has already been found in different environments, such as sludge samples from anaerobic reactors used in effluent treatment [3,5], landfill soil [6], biogas plant reactors [7] and seawater [8]. *O. teriensis* is a Gram-negative, anaerobic, rod-shaped bacteria with a structure at the ends called a ‘toga’ with multiple flagella. It is moderately thermophilic and grows in a wide range of salinity. It can use thiosulfate and elemental sulphur with the final electron acceptor during the respiration process, releasing hydrogen sulfide (H_2_S) as the end product of the reaction [1]. This sulfidogenic metabolism can significantly accelerate the metallic corrosion of oilfield installations, becoming a problem for the sector [9]. Previous work has shown that the corrosion rates promoted by thiosulfate-reducing bacteria (TRB) are similar to the corrosion rates advertised by sulfate-reducing bacteria (SRB) [10,12]. SRBs are the leading group of bacteria causing microbiologically induced corrosion (MIC) [13]. However, little is known about the characteristics of this microbial species, genomic diversity and antibiotic resistance.

The emergence and spread of antibiotic resistance genes (ARGs) among pathogenic bacteria, in addition to the emergence of multidrug-resistant bacteria, have been significant public health concerns in recent decades worldwide. Estimates around 1.27 million deaths were directly attributed to antimicrobial resistance (AMR) in 2019, with the number of AMR-associated deaths being four times higher at about 4.95 million [14]. The emergence of multidrug-resistant bacteria has been accelerated due to the excessive and inappropriate use of antibiotics in treating diseases and in animal husbandry [15].

Recently, it has been shown that environments affected by offshore oil exploration show an increase in the abundance of sulfonamide resistance genes through horizontal gene transfer (HGT), which may be a risk to public health [16]. Knowing this, physical contact between domestic sewage and wastewater from offshore oil exploration allows contact between pathogenic bacteria found in the gastrointestinal system and non-pathogenic multi-resistant bacteria, facilitating the genetic exchange of ARGs between them.

The HGT is one of the main reasons for the spread of ARGs in the environment [17]. This transfer can occur through three different mechanisms, which include transformation, conjugation and transduction [18]. Environmental bacteria are considered reservoirs of ARGs [19,20]; therefore, the identification and monitoring of potential risks of horizontal transfer of ARGs are extremely important.

Bacteriophages facilitate gene transfer through transduction [21]. Given this, bacteria have developed a series of defence mechanisms against infection and death by phages in response to the rapid evolution of phage particles, triggering an evolutionary arm race. The restriction–modification (RM) system, CRISPR (clustered regularly interspaced palindromic repeats) and the abortive infection (ABI) system are the best-studied microbial defence mechanisms against phages [22]. CRISPR and their associated genes (*cas*) make up the adaptive immune system, which provides prokaryotes with protection against invasion by viral sequences, plasmids and other mobile genetic elements [23]. The search for CRISPR arrays makes it possible to determine phage resistance profiles since it is already known that the content of spacers is chronologically associated with bacteriophage infection [24].

Due to the small number of studies on AMR and the presence of prophages and phage infection immunity systems in bacteria related to the metallic corrosion process in offshore oil exploration platforms, it is of great interest to determine the antimicrobial susceptibility profile, in addition to understanding the involvement of bacteriophages and other mobile elements in the diversity of this microbial group, for the future development of bacterial control methods, since such environmental bacteria are considered a reservoir of mobile genetic factors, being a concern for the health system, as it can accelerate the emergence of multidrug-resistant bacteria.

In this work, we carried out comparative genomic analysis, evaluation of susceptibility to antibiotics, search for genes for resistance to antibiotics and heavy metals, mobile genetic elements and systems of resistance to phage infection in the strain *O. teriensis* UFV_LIVM02 isolated from a sample from the production water separator of an oil extraction platform in the Campos Basin, Rio de Janeiro, Brazil. By annotating the genome, it was possible to observe the presence of resistance genes to beta-lactams and multidrug efflux pumps. However, *in silico* detection of ARGs using the Comprehensive Antibiotic Resistance Database (CARD) revealed numerous resistance genes, considering only hits with weak similarity (called Loose), against AMRs deposited in the databases, which may represent new previously uncharacterized AMRs, since there are no reports in the literature that searched for resistance genes for the species of *O. teriensis*. Furthermore, the *in silico* presence of five genomic islands was verified, with a varied number of genes, nine immunity systems to phage infection, four CRISPR arrays with a variety of spacers and the absence of prophages in their genome.

## Methods

### Isolation

The UFV_LIMV02 strain was isolated from a production water separator sample from an oil extraction platform in the Campos Basin, Rio de Janeiro, Brazil. This strain was cultivated in a modified Postgate C medium pH 7.2 (Table S1, available in the online version of this article) and incubated at 30 °C in anaerobic conditions for about 4 days. After growth, the culture was seeded in the same solid medium [plus 1.5 % (w/v) of agar] and incubated at 30 °C in an anaerobic chamber (mixture of N_2_ : H_2_:CO_2_ gases; 80 : 10 : 10), until the emergence of colonies. The isolated colonies were seeded again on a solid medium to obtain a pure culture; after repeating the procedure three times, the isolated colonies were transferred anaerobically to a liquid medium and incubated as described above.

### Growth curve and transmission electronic microscopy

The culture was inoculated into Hungate culture tubes with 9 ml of modified Postgate C medium and incubated at 30 °C for 6 days. The optical density was monitored daily, with three readings per day, during all days of analysis, using a DR3900 tube spectrophotometer (Hach, Ames, IA, USA). All analyses were performed in triplicate. The morphology of the isolate was visualized by transmission electron microscopy (TEM). A 10-µl aliquot of the bacterial suspension was deposited on 200 mesh grids coated with FormVar (Sigma) for negative staining. After removing excess liquid, 2 µl of 2 % uranyl acetate solution was added to the grid. The samples were then visualized using a Zeiss EM 109 electron microscope operating at 80 kV at the Nucleus of Microscopy and Microanalysis of the Federal University of Viçosa-UFV (Viçosa, Minas Gerais, Brazil).

### Amplification and analysis of the 16S rRNA gene

The extracted genetic material was subjected to a polymerase chain reaction (PCR) using the Veriti TM 96-Well thermocycler (Applied Biosystems, Foster City, CA, USA). For amplification of the 16S rRNA gene, primers 10 f (5′-AGTTTGATCCTGGCTCAG-3′) and 1100 r (5′-GGGTTGCGCTCGTTG-3′) were used [25]. Approximately 100 ng of DNA was added to GoTaq Green Master Mix (Promega, Madison, WI, USA), along with 1 µl of each primer (10 µM), and subjected to the following cycling conditions: initial denaturation at 95 °C for 5 min, 35 cycles of denaturation at 95 °C for 1 min, annealing at 57 °C for 1 min and extension at 72 °C for 1 min. A final extension was performed at 72 °C for 5 min. Amplicons were purified using the Promega Wizard SV Gel and PCR Clean-Up System kit (Promega, Madison, WI, USA) following the manufacturer’s recommendations and sent for sequencing. The sequence obtained was compared with those deposited in the GenBank database (http://www.ncbi.nlm.nih.gov) using the BLASTn platform (https://blast.ncbi.nlm.nih.gov/Blast.cgi). The sequences were aligned and analysed with the mega 11 software [26]. Subsequently, the Neighbor-Joining method constructed a phylogenetic tree with 1000 × Bootstrap.

### Genomic DNA extraction

Genomic DNA was extracted using the standard phenol/chloroform extraction method followed by ethanol precipitation [27]. DNA purity was verified by 1 % (w/v) agarose gel electrophoresis. DNA quality and concentration were evaluated using Nanodrop 2000 (Thermo Fisher Scientific, Wilmington, DE, USA) and Qubit 4 (Life Technologies, Invitrogen, Darmstadt, Germany), respectively.

### Genome sequencing, assembly and annotation

Novogene Inc. (Sacramento, CA) conducted library preparation and sequencing using the Illumina NovaSeq 6000 platform. To assess the quality and detect possible problems in the sequencing datasets, FastQC version 0.11.9 (https://github.com/sandrews/FastQC) was used. Then, the TrimGalore automatic detection configuration was used (version 0.6.7) (https://github.com/FelixKrueger/TrimGalore). Trimmomatic version 0.36 was used to filter the high-quality reads with the following parameters: HEADCROP:10 CROP:140 SLIDINGWINDOW:4 : 20 MINLEN:130 [28]. Subsequently, the filtered reading frames were assembled by the *de novo* method, using SPAdes version 3.15.4, selecting all odd k-mers between 21 and 99 [29]. The Assembly-Stats programme version 1.0.1 (https://github.com/sanger-pathogens/assembly-stats) analysed the assemblies, excluding contigs smaller than 1000 bp. The Rapid Annotation using Subsystem Technology (RAST) programme (http://rast.nmpdr.org/rast.cgi) [30] and the National Centre for Biotechnology Information (NCBI) Prokaryotic Genome Annotation Pipeline (PGAP) [31] were used to annotate the coding DNA sequences (CDSs). The UFV_LIMV02 genome has been deposited in the NCBI GenBank database accession number JAMHJO000000000.1, BioProject PRJNA837603, BioSample SAMN28208037 and assembly accession number GCF_030587775.1.

The UFV_LIVM02 genome is the second deposited genome of the species *O. teriensis* at NCBI; therefore, a comparative genomic analysis of strain UFV_LIMV02 and strain DSM 24906 (GenBank accession number NZ_QGGI00000000.1) was performed using Progressive Mauve [32], following the default settings. Average nucleotide identity (ANI) and digital DNA–DNA hybridization (dDDH) were used to validate species identity, using JSpecies software [33] and Genome-to-Genome Distance Calculator web service (https://www.dsmz.de/services/online-tools/genome-to-genome-distance-calculator-ggdchttps://ggdc.dsmz.de/ggdc.php#).

### Antimicrobial sensitivity test

The antimicrobial sensitivity assay was performed using the disc diffusion method (DME Sensidisc, Araçatuba, SP, Brazil). First, the bacterial isolate was cultured in 10 ml of modified Postgate C liquid medium at 30 °C, under anaerobic conditions, to an OD_600_ of 0.1. Then, 1 ml of the bacterial suspension was added to 9 ml medium plus 0.7 % (w/v) of agar and spread on the culture plate already containing solid medium; the discs were placed on the surface of the plate and then incubated at 30 °C for 4 days in anaerobic conditions. Altogether, 20 different antibiotics were analysed and distributed into 11 classes (Table S2). The *Escherichia coli* ATCC 25922 strain was used as quality control of the antibiotics used in the antimicrobial susceptibility test.

### Identification of AMR genes

ARGs were annotated rapidly online using subsystem technology (RAST) 2.0, but this search was expanded using ResFinder v4.5.0 [34] and the CARD programme (https://card.mcmaster.ca/) [35], keeping ‘Loose’ correct.

### Prediction of mobile genetic elements and antiphage defense systems

The IslandViewer web server version 4 was used to forecast the existence of genomic islands [36]. To predict prophage regions, the web server PHASTEST (PHAge Search Tool with Enhanced Sequence Translation) [37]. Antiphage defence systems have been predicted with both DefenseFinder [38] and the Prokaryotic Antiviral Defence Locator (PADLOC) [39]. The CRISPRDetect algorithm [40] was used to find CRISPR regions in the genome sequence.

## Results and discussion

### Growth curve and morphology UFV_LIMV02

**Fig. 1.**
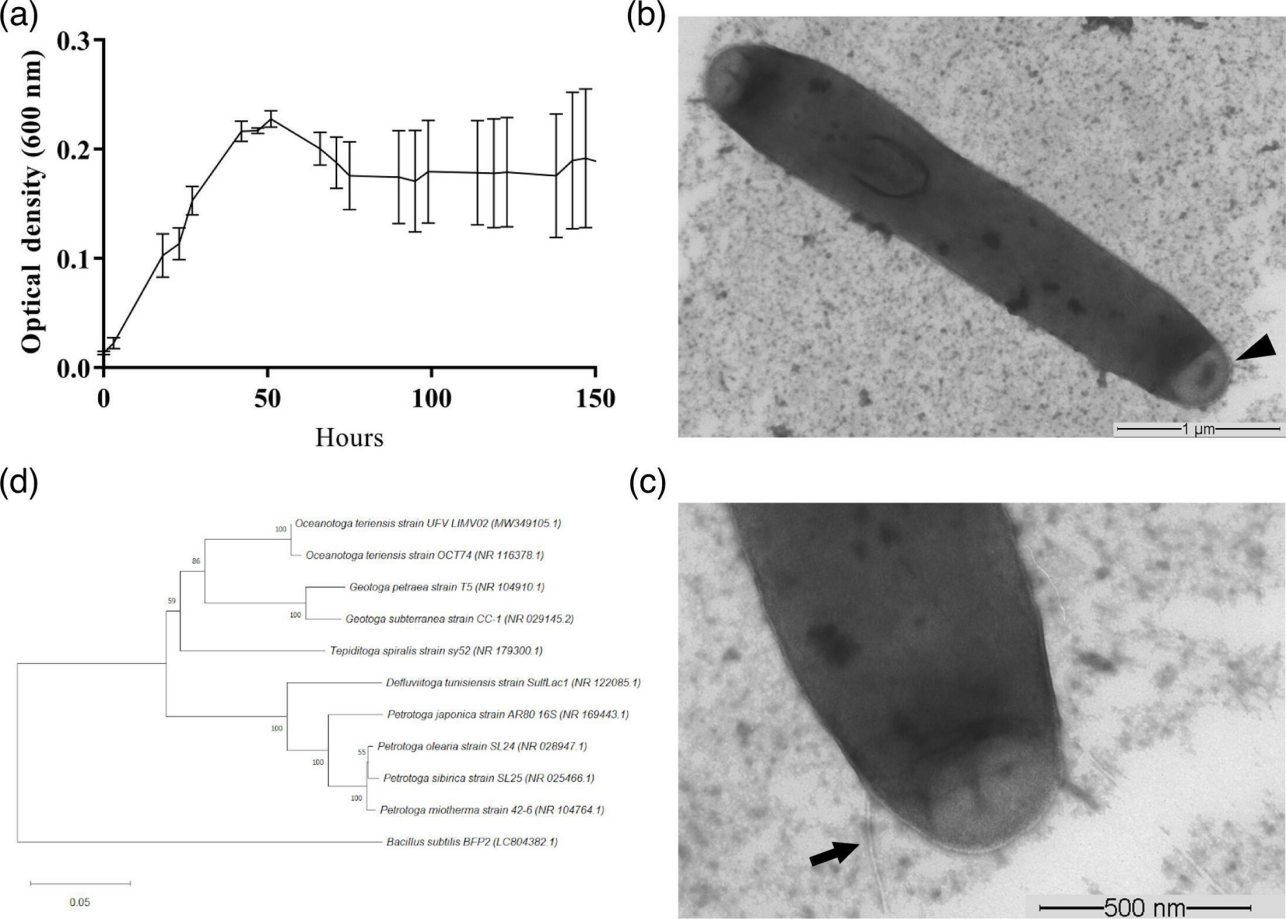
**(a**) Growth curve by *O. teriensis* strain UFV_LIMV02 for 6 days. (**b**) TEM showing morphological characteristics of the bacterial cell. The arrowhead is the ‘toga’, a specific structure of the order *Thermotogales*. Bar=1 µm. (**c**) In TEM magnification, the arrow indicates the flagellum. Bar = 500 nm. (**d**) Phylogenetic tree based on the partial sequence of the 16S rRNA of the isolates UFV_LIVM02 (*O. teriensis*), marked with an asterisk. The tree was built using the Neighbor-Joining method with Bootstrap value represented in the branches for 1000 repetitions. The sequence accession number in the GenBank is shown in parentheses.

The bacterial growth of isolate UFV_LIMV02 was monitored by measuring the optical density at 600 nm of the bacterial liquid culture for 6 days, with three readings during the day (Fig. 1a). The curve was started with a density of 0.01 in 10 ml of liquid medium. When analysing the growth curve, it is possible to observe that *O. teriensis* presented an exponential growth during the first 51 h of cultivation, reaching the maximum OD_600_ of 0.227 ± 0.006. When the bacteria reached maximum optical density, there was a slight drop in the OD_600_ during the following 24 h of analysis; after 75 h of cultivation, it reached the stationary phase and remained until the end point of the study of 144 h.

Through TEM, it was possible to observe the morphology of the cells of the UFV_LIMV02 strain. The cells showed a rod shape, with a diameter of 0.64 µm and a length of 3.8 µm (Fig. 1b), containing an outer envelope at both ends of the cells in a sheath shape called a toga. This structure is characteristic of the members of *Petrotogales* [2]. This layer serves as a barrier to the external environment but also allows the organization of polysaccharide hydrolase on the cell surface, favouring insoluble carbon sources [41,42]. In addition, TEM images revealed the presence of flagella, as indicated by the arrow (Fig. 1c).

### Identification and phylogenetic analysis using the 16S rRNA

The phylogenetic analysis using the 16S rRNA gene confirms the taxonomic identification of the isolate (Fig. 1d). The alignment of the sequences of the isolate with the sequences of the GenBank database revealed that the UFV_LIMV02 isolate is related to members of the genus *Oceanotoga* with 100 % identity with the species *O. teriensis* (accession number EU588727.1). The 16S rRNA partial sequence of *O. teriensis* UFV_LIMV02 was deposited in the NCBI database with accession number MW349105.1.

### Genomic assembly and annotation

The final assembly of the *O. teriensis* UFV_LIMV02 genome resulted in 34 scaffolds, with a total length of 2 812 289 bp, an average G+C content of 26 %, an N50 value of 1 62 Please clear this DTD va472 bp and L50 of 5 bp. Identification of coding sequences and primary annotation were performed using the NCBI PGAP version 4.7, identifying 2700 genes, 2649 protein-coding sequences, 15 pseudogenes and 51 functional RNA genes, with five rRNA-coding genes, 43 tRNA-coding genes and three ncRNA-coding genes. This genome was the second genome of the species *O. teriensis* sequenced, and a comparison of the general characteristics of the genome of the strain UFV_LIMV02 and another genome of the species is summarized in Table 1.

**Table 1.** Comparison of the genomic characteristics of species *O. teriensis*

Features	*O. teriensis* UFV_LIMV02	*O. teriensis* DSM 24906
Accession No.	JAMHJO000000000.1	NZ_QGGI00000000.1
Genome size (bp)	2 812 489	2 792 290
G+C content (%)	26.0	25.9
No. of contigs	34	45
N50 length (bp)	162 472	95 346
L50	5	9
Genes	2700	2683
CDSs (total)	2649	2629
tRNA	43	43
rRNA	5	8
CRISPR system	4	5

Based on the NCBI PGAP.

**Fig. 2.**
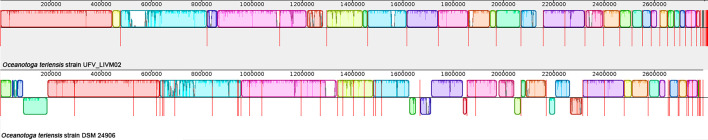
Genome synteny analysis. Schematic of genome alignment obtained using Mauve software package. The study was performed using the genomic sequence of the isolate UFV_LIVM02 (JAMHJO000000000.1) and the reference genome DSM 24906 (NZ_QGGI00000000.1), and blocks with the same colour represent homologous regions. Blocks illustrated above the X-axis are on the positive strand (forward), while the blocks below the X-axis are on the negative strand (reverse), being the genes that undergo the inversion.

Alignment of the complete genomic sequence of the isolate with the DSM 24906 strain revealed high levels of synteny between the genomes. However, some divergences were observed, such as inversions in some genome regions, as shown in Fig. 2. The comparison of nucleotide identity between the genomes of the UFV_LIMV02 strain and the DSM 24906 strain indicated a high similarity, as indicated by the same ANIb at 99.33 % and ANim equal to 99.54 %, which is higher than the conventional cut of 95 % for species delimitation [43]. To quantitatively assess the genomic diversity between the two strains of *Oceanotoga*, the digital dDDH was calculated, which uses established limits to delineate species DDH >70 % and subspecies DDH >79 % [44,45]. The analysis revealed a cutoff value of 97.59 % for the species design and 75.2 % for the subspecies. This result agrees with the result found for ANI; the isolate UFV_LIMV02 should be considered a species of *O. teriensis*. Although the dDDH for subspecies of UFV_LIMV02 was below the 79 % limit delineating subspecies, future studies are needed to verify whether the isolate in question is truly a subspecies of *O. teriensis*. Genotypic and phenotypic characterization is necessary to determine whether an isolate is a subspecies of an already described species. One of the phenotypic analyses is the comparison of cell morphology. Compared to the only morphologically described isolate *O. teriensis* OCT74^T^ [1] and UFV_LIMV02 (Fig. 1b), we can verify some distinctions, such as the size and presentation of the toga in the cells. This is an indication that the isolate UFV_LIMV02 may be a subspecies of *O. teriensis*, but future analyses and standardization of cultivation conditions are necessary so that we can have more solid evidence.

The functional analysis was performed with the RAST server, which predicted a total of 2716 genes, 2669 CDSs and 47 RNA genes. Most of the protein-coding genes (77.93 %) received putative functions, and the others were annotated as hypothetical proteins. A total of 1369 genes were distributed in 27 categories, the most abundant being related to ‘carbohydrate’ metabolism with 341 genes, followed by ‘protein metabolism’ with 218 genes, ‘amino acids and derivatives’ with 165 genes, ‘cofactors, vitamins, prosthetic groups, pigments’ with 146 genes, ‘RNA metabolism’ with 104 genes and ‘cell wall and capsule’ with 95 genes (Fig. S1). Among all genes assigned to a category in RAST, a significant portion (~25 %, 341 genes) is dedicated to carbohydrate utilization, which is in line with its versatile use of different carbon sources, as shown by Jayasinghearachchi and Lal [1].

### The drug resistance phenotype and genotype analysis of the UFV_LIMV02

The antimicrobial susceptibility profile of *O. teriensis* UFV_LIMV02 was evaluated by disc diffusion assays; a total of 20 antibiotics belonging to 11 categories were analysed to test the antimicrobial phenotype of the isolated strain. The results show that UFV_LIMV02 (Table 2) was resistant to most of the antibiotics evaluated. There was no change in growth in the presence of all beta-lactams (cefepime, ceftriaxone, ceftazidime, cefoxitin, amoxicillin/clavulanic acid, ampicillin, piperacillin/tazobactam and aztreonam), aminoglycosides (gentamicin and amikacin), lincosamide (clindamycin), fluoroquinolones (ciprofloxacin), sulfonamides (sulfamethoxazole/trimethoprim) and rifamycins (rifampicin). Only six antibiotics were able to inhibit growth, presenting inhibition zones between 25 and 40 nm, namely amphenicols (chloramphenicol), tetracyclines (tetracycline), macrolides (azithromycin and erythromycin) and glycopeptides (vancomycin) and oxazolidinones (linezolid).

**Table 2.** Antibiotic susceptibility profiling of *O. teriensis* UFV_LIVM02 by disc diffusion method

Class	Antibiotic*	ID	Dose (µg)	Zone of inhibition (mm) *	Interpretation
Beta-lactam	Cefepime	CPM 30	30	0	R
	Ceftriaxone	CRO 30	30	0	R
	Ceftazidime	CAZ 30	30	0	R
	Cefoxitin	CFO 30	30	0	R
	Amoxicillin/clavulanic acid	AMC 30-20/10	30	0	R
	Ampicillin	AMP 10	10	0	R
	Piperacillin/tazobactam	PIT 110	110	0	R
	Aztreonam	ATM 30	30	0	R
Macrolides	Azithromycin	AZI 15	15	30	S
	Erythromycin	ERI 15	15	40	S
Aminoglycosides	Gentamycin	GEN 10	10	0	R
	Amikacin	AMI 30	30	0	R
Lincosamides	Clindamycin	CLI 02	02	0	R
Amphenicols	Chloramphenicol	CLO 30	30	25	S
Tetracyclines	Tetracycline	TET 30	30	33	S
Fluoroquinolones	Ciprofloxacin	CIP 05	05	0	R
Glycopeptides	Vancomycin	VAN 30	30	30	S
Oxazolidinones	Linezolid	LNZ 30	30	30	S
Sulfonamides	Sulfamethoxazole/trimethoprim	SUT 25	25	0	R
Rifamycin	Rifampin	RIF 05	05	0	R

*The ranges of inhibition zones were calculated from three individual replicates, and ‘0’ means no inhibition zone.

Among the subsystems, RAST identified 58 genes associated with virulence, diseases and the organism’s defence (Fig. S1). A relevant number of genes related to resistance to antibiotics and toxic compounds were identified, a total of 44 genes, representing 3.21 % of the reads annotated by the SEED subsystems; these data are available in Table 2. Resistance to antibiotics and toxic compounds is essential for microbial survival and adaptation in contaminated environments. Genes that confer resistance to beta-lactams, macrolides and heavy metals such as copper, zinc, cobalt and cadmium were identified. Also, a gene that makes up the multidrug efflux pump system (Multidrug Resistance Efflux Pumps) was detected (Table 3); this system allows bacteria to expel antibiotics of different classes from their cytoplasm, thus reducing their antimicrobial action [46].

**Table 3.** Summary of protein-coding sequences annotated by RAST as belonging to the group of genes ‘resistance to antibiotics and toxic compounds’

Subcategory	Role	Scaffold
Resistance to antibiotics and toxic compounds	Copper-translocating P-type ATPase (EC 3.6.3.4)	2, 6, 14, 14
	Cobalt-zinc-cadmium resistance protein	6
	Probable Co/Zn/Cd efflux system membrane fusion protein	8
	Transcriptional regulator, MerR family	1, 6, 7, 8, 14, 21, 22
	Streptothricin acetyltransferase, Streptomyces lavendulae type	16
	Copper homeostasis protein CutE	27
	Magnesium and cobalt efflux protein CorC	3
	DNA gyrase subunit B (EC 5.99.1.3)	10
	DNA gyrase subunit A (EC 5.99.1.3)	1
	Beta-lactamase class C and other penicillin-binding proteins	2, 2
	Beta-lactamase (EC 3.5.2.6)	1, 1, 22
	Cadmium efflux system accessory protein	2, 4
	Multi antimicrobial extrusion protein (Na (+) /drug antiporter), MATE family of MDR efflux pumps	1, 1, 1, 2, 2, 4, 4, 5, 6, 7, 9, 9, 14, 18, 21, 23, 24, 28
	Macrolide export ATP-binding/permease protein MacB (EC 3.6.3.-)	8

The antibiotic resistance observed for the eight different beta-lactam antibiotics tested can be explained by the presence of two beta-lactamases in its genome, allowing its growth when exposed to these antimicrobials. Resistance to the antibiotics gentamicin, amikacin and ciprofloxacin may be related to the presence of multidrug efflux transporters belonging to the multidrug and toxic compound extrusion (MATE) family, as previous work has already revealed [47,48]. Through RAST Annotation, no genes that confer resistance to the antibiotics clindamycin, sulfamethoxazole/trimethoprim and rifampicin were detected.

Strain UFV_LIMV02 was isolated from a sample of production water from an oil extraction platform. Generally, wastewater from oil exploration is characterized by high concentrations of heavy metals [49]. The presence of these metals in wastewater may contribute to the prevalence of heavy metal resistance genes, contributing to microbial selection. Studies report increased antibiotic resistance in microbial communities exposed to heavy metals, establishing a theory of co-sealing of genes associated with resistance to metals and antibiotics in environments contaminated by heavy metals [50,52].

Co-selection of microorganisms with antibiotic and heavy metal resistance genes has been a concern since the 1970s [53]. This process refers to the phenomenon where the presence or exposure to one selective agent, such as an antibiotic, can lead to increased prevalence or maintenance of resistance genes to another unrelated selective agent, such as heavy metals. In the context of bacterial resistance, co-selection often occurs due to the proximity of genes that confer resistance to antibiotics and heavy metals on mobile genetic elements like plasmids, integrons, prophages or transposons. These elements can be transferred between bacteria through HGT mechanisms like conjugation, transformation or transduction [54]. Therefore, a major concern for the medical community, as industrial and agricultural effluents loaded with metals are often released into the environment, contributes to the maintenance and transfer of ARGs in pathogens [55]. The acquisition of ARGs can occur through HGT; this genetic exchange can occur by different mechanisms, whether by transformation, transduction by bacteriophages or conjugation involving plasmids [56,57]. Regardless of the means of resistance acquisition, this problem has been intensified by the widespread and indiscriminate use of antibiotics by humans in recent years, which has accelerated the emergence of resistant and multidrug-resistant bacterial strains, not only among human pathogens but also in environmental bacteria [14,58].

The search for AMR genes *in silico* in the genome of the isolate UFV_LIMV02 using ResFinder did not identify any ARGs. When we analysed the genome using the CARD, no gene was identified as 'Perfect Hit'; however, a total of 198 genes were found, with low similarity (called Loose), which may provide the detection of new genes (Table S3), requiring future studies. A total of 31 different classes of drugs were found, distributed across six different resistance mechanisms: alteration of the antibiotic target, antibiotic inactivation, antibiotic efflux, replacement of the antibiotic target, reduced antibiotic permeability and antibiotic target protection (Table S3). The antibiotic efflux pump category represented the majority of hits to all those analyzed. Due to their possible link to clinical multidrug resistance, multidrug efflux pumps have drawn special attention recently [58]. Furthermore, Wang and Zhou showed in their work the presence of genes related to the transport of multidrug in sediment samples adjacent to an oil exploration platform, suggesting that offshore oil exploration favours the spread of antibiotic resistance, through the HGT of ARGs [59]. It is believed that bacteria, when exposed to constant physicochemical changes in their habitat, present better adaptability and competitive aptitude to overcome the environmental challenges encountered, which can lead to the acquisition of ARGs [60].

Although no ARG was detected with high reliability using the ResFinder and CARD programmes, it was verified through the antibiogram that the growth of the UFV_LIVM02 isolate was inhibited by 14 different antibiotics out of the 20 analysed, which attracted attention. Both programmes use well-established and widespread ARG databases. This limits the detection of new ARGs, as they are typically not recognized until they are widely publicized. Previous work has shown that offshore oil exploration environments have an abundance of ARGs [16,59, 61], thus favouring the emergence of non-pathogenic bacteria that are multiresistant to different antimicrobials, as we verified with UFV_LIVM02. Environmental bacteria harbour a diverse set of ARGs, which can over time be mobilized and transferred to pathogens [62].

### Identification of mobile genetic elements in *O. teriensis* isolate

**Fig. 3.**
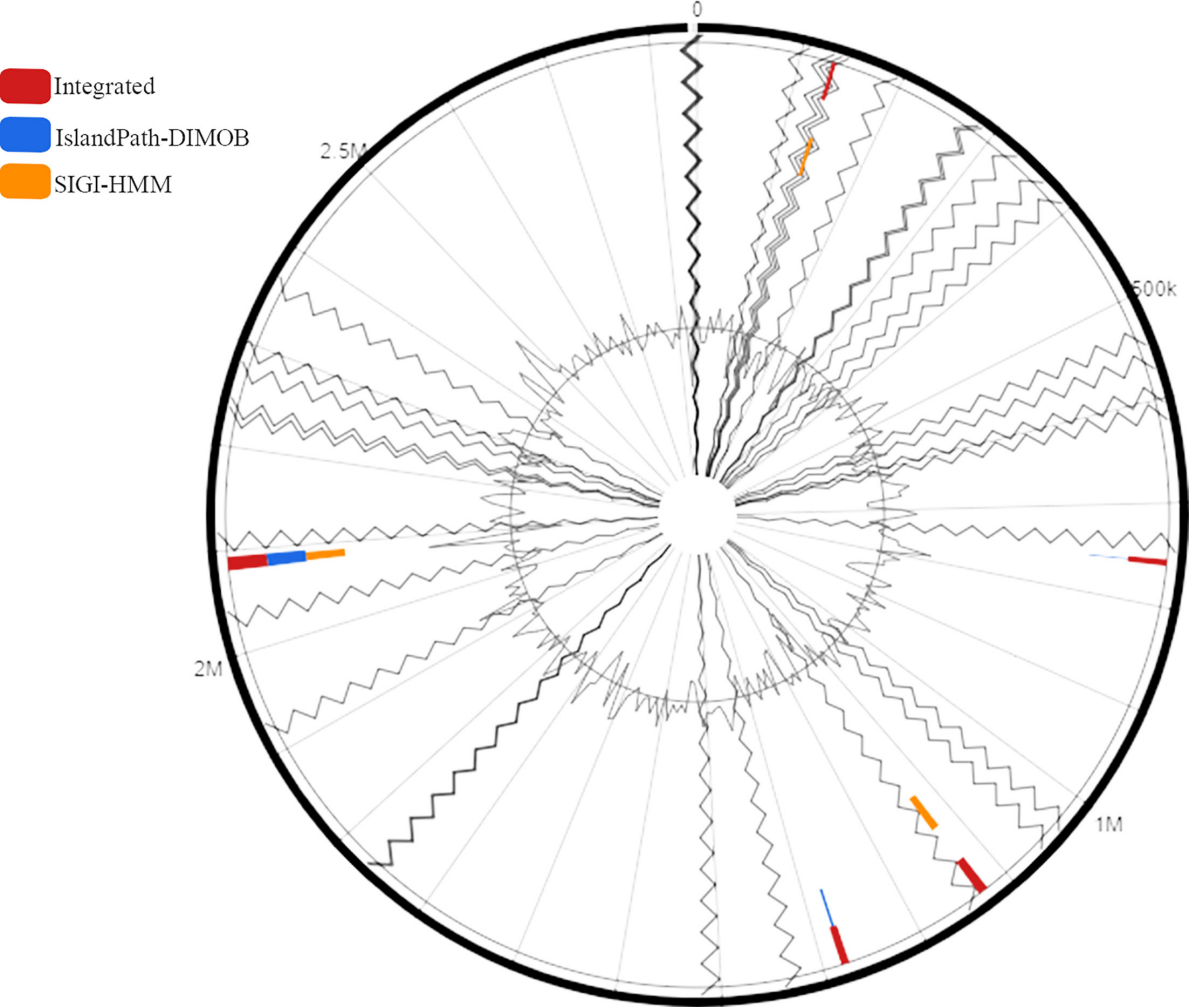
Circular map generated by IslandViewer 4.0 representing the location of genomic islands within the genome of *O. teriensis* UFV_LIVM02, when aligned against the complete genome of *Oceanotoga* sp. DSM 15011 as reference. The outer black circle represents the scale line in Mbps, the black zigzag line delineates each of the 34 contigs and the black graph represents GC content (%). Orange bars represent genomic islands (GIs) identified through the SIGI-HMM genomic island prediction software, blue bars are GIs identified through the IslandPath-DIMOB programme and integrated GIs identified through all programmes used are represented by red bars.

Prediction of genomic islands using IslandViewer version 4.0 revealed the presence of a total of five genomic islands within the *O. teriensis* UFV_LIVM02 genome when aligned to the *Oceanotoga* sp. DSM 15011 reference genome, as depicted in Fig. 3. Two different methods predicted the genomic islands, and the colour represents the prediction method: IslandPath-DIMOB (blue), SIGI-HMM (orange) and integrated results (dark red). These genomic islands have varying lengths, between 4612 and 14 076 kb, and each island contains between 6 and 14 genes. However, a substantial portion, approximately 64.44 % of the total genomic island genes (29 of 45), remained functionally uncharacterized, being described as hypothetical proteins. These sequences were subjected to similarity analysis using HMMER using the UniProt database and annotated as shown in Table 4.

**Table 4.** List of genes present in the genomic islands of *O. teriensis* UFV_LIVM02 identified by IslandViewer 4. Gene function was annotated via RAST; any hypothetical or uncharacterized proteins were further analysed via HMMER using the UniProt database

Island	Function	Annotation (organism)	Length (aa)
1	Hypothetical protein	HTH-type transcriptional activator mta (*Bacillus subtilis* strain 168)	188
	ABC transporter ATP-binding protein		270
	**ABC-type multidrug transport**		**241**
	Hypothetical protein	Not determined	243
	Hypothetical protein	Not determined	54
	Hypothetical protein	ADP-d-ribose sintase cíclica putativa ThsB1 (*Bacillus cereus* strain MSX-D12)	163
2	Mobile element protein	Insertion sequence IS5376 putative ATP-binding protein (*Geobacillus stearothermophilus*)	253
	Mobile element protein	Transposase (*Aminobacter aminovorans*)	514
	Hypothetical protein	Not determined	271
	Hypothetical protein	Not determined	60
	Hypothetical protein	Not determined	41
	Hypothetical protein	Not determined	83
	Hypothetical protein	Not determined	105
	Methyl-accepting chemotaxis protein		477
	Translation elongation factor P		187
3	Hypothetical protein	Uncharacterized protein YlaK (Geobacillus stearothermophilus)	145
	Hypothetical protein	CRISPR system ring nuclease SSO1393 (*Saccharolobus solfataricus* strain ATCC 35092)	377
	Hypothetical protein	Not determined	158
	Hypothetical protein	Not determined	125
	Hypothetical protein	Not determined	40
	Hypothetical protein	Not determined	145
	Hypothetical protein	RNA polymerase sigma factor SigA (*Thermotoga maritima* strain ATCC 43589)	877
	Hypothetical protein	Not determined	156
4	Cell division protein FtsH (EC 3.4.24.-)		620
	Methyl-accepting chemotaxis protein		237
	Mobile element protein	Putative transposase InsK for insertion sequence element IS150 (*Escherichia coli* strain k12)	265
	ISSth1, transposase (orf1), IS3 family		237
	Methyl-accepting chemotaxis protein		431
	Hydrolase, haloacid dehalogenase-like family		222
	DEAD-box ATP-dependent RNA helicase CshA (EC3.6.4.13)		588
	Transcriptional regulator, AbrB family		75
5	**Transcriptional regulator, MerR family**		115
	**Copper-translocating P-type ATPase (EC3.6.3.4)**		73
	ISSth1, transposase (orf1), IS3 family	Probable transposase for insertion sequence element IS5377 (*Geobacillus stearothermophilus*)	253
	Mobile element protein	Putative transposase InsK for insertion sequence element IS150 (*Escherichia coli* strain k12)	218
	**Lead, cadmium, zinc and mercury transporting ATPase (EC 3.6.3.3) (EC 3.6.3.5); copper-translocating P-type ATPase (EC 3.6.3.4**)		657
	**Hypothetical protein**	**UPF0236 protein in vanSb 3' region** (***Streptococcus gallolyticus***)	**472**
	**Transcriptional regulator, TetR family**		**194**
	Hypothetical protein	Not determined	149
	Transposase		445
	Mobile element protein	Transposase for insertion sequence element IS1001 (*Bordetella parapertussis*)	449
	**Transcriptional regulator, MerR family**		**156**
	Radical SAM domain protein		399
	Endonuclease IV		303
	DNA-3-methyladenine glycosylase (EC 3.2.2.20)		186

Annotated antibiotic and heavy metal resistance genes are highlighted in bold.

Genomic island 1 contains six annotated genes, including one gene encoding a protein involved in gene regulation in the bacterium *Bacillus subtilis*, a signalling protein of the Thoeris antiviral defence system [63], an ABC-type transporter protein and an ABC-type multidrug transport system protein; such a protein gives bacteria the ability to evade most current therapies [64]; two genes were annotated with hypothetical proteins, and it was not possible to functionally characterize these proteins. On genomic island 2, two mobile genetic elements were identified; the first was an insertion sequence (IS) originating from *Geobacillus stearothermophilus*, a thermophilic bacterium generally found in oil wells or places contaminated with oil [65,66], when compared with isolate UFV_LIVM02, both occupy the same niche. The second mobile element is the gene encoding the transposase of *Aminobacter aminovorans*, a bacteria present in the soil [67]. Also on this island, a gene encoding a protein associated with chemotaxis in bacteria and protein translation was found. And four hypothetical proteins have not been characterized until now.

On island 3, most genes encode proteins annotated with hypothetical or uncharacterized proteins, except for a nuclease belonging to the CRISPR system from *Saccharolobus solfataricus* and an RNA polymerase from *Thermotoga maritima*, both described in thermophilic microorganisms. The fourth island contains two genes related to chemotaxis, one gene responsible for the synthesis of hydrolase, two genes related to the transcription and translation process and, lastly, two transposases, one of which comes from *E. coli*, a bacterial species usually found in the gastrointestinal tract of humans and endothermic animals.

Finally, the fifth island, being the largest genomic island found in UFV_LIVM02, is composed of three proteins involved in DNA repair: radical SAM [68], endonuclease IV [69] and DNA-3-methyladenine glycosylase [70]. There are four transposases, one of which again originates from *E. coli*, one from *Bordetella parapertussis*, a bacterium considered a human pathogen, which colonizes the respiratory tract, causing whooping cough [71]; another transposase originates from the species *G. stearothermophilus*, and the last one has not been determined. Four membrane fusion proteins of the different metal efflux system (Pb/Cd/Zn/Co/Zn/Hg) and two genes related to the antibiotic resistance process, the first is vanB, are associated with vancomycin resistance, and another gene encodes the transcriptional regulator of the tetracycline repressor protein (TetR) family. This protein controls the expression of the tet genes, whose products confer resistance to tetracycline [72]. These genes may play a role in the AMR of * O. teriensis* UFV_LIVM02. According to the data shown in genomic island 5 (Table 4), we can confirm the theory of co-selection of resistance genes and heavy metals in the isolate analysed. Both genes make up the same genomic island and are located near four different transposons. These genes were probably transferred between bacteria through HGT mechanisms.

An important point that the analysis of genomic islands revealed is the presence of mobile elements originating from human pathogens such as *E. coli* and *B. parapertussis*. Therefore, a concern arises on the transfer of ARGs between environmental bacteria and intestinal bacteria with potential pathogens in the waste treatment plants of offshore oil exploration platforms in the extraction of oil, and the domestic sewage from the platforms is mixed and treated in the same sewage treatment plant. This would favour gene exchange between environmental bacteria and bacteria in the intestinal tract, knowing that many bacteria present in the intestinal microbiota have different genetic elements that help in the acquisition of genes and their transfer to pathogens [73].

No prophage region was found within the genomic islands, but lysogenic phages are part of the mobile genetic elements present in bacteria; therefore, they are effective vectors for the acquisition and dissemination of ARGs [74]. Possible prophage regions incorporated into the *O. teriensis* UFV_LIMV02 genome were searched using PHASTEST. The results showed that no phage region of the UFV_LIVM02 genome was observed.

### Antiphage systems

The ecology and evolution of microbial communities depend heavily on the interactions between bacteria and phages. To thwart phage attacks and lysis, bacteria have developed a variety of antiphage systems, including retrons, CRISPR–Cas systems, restriction modification systems and toxin-antitoxin (TA) [75]. These systems prevent phage infection at various stages of the infection cycle.

**Fig. 4.**
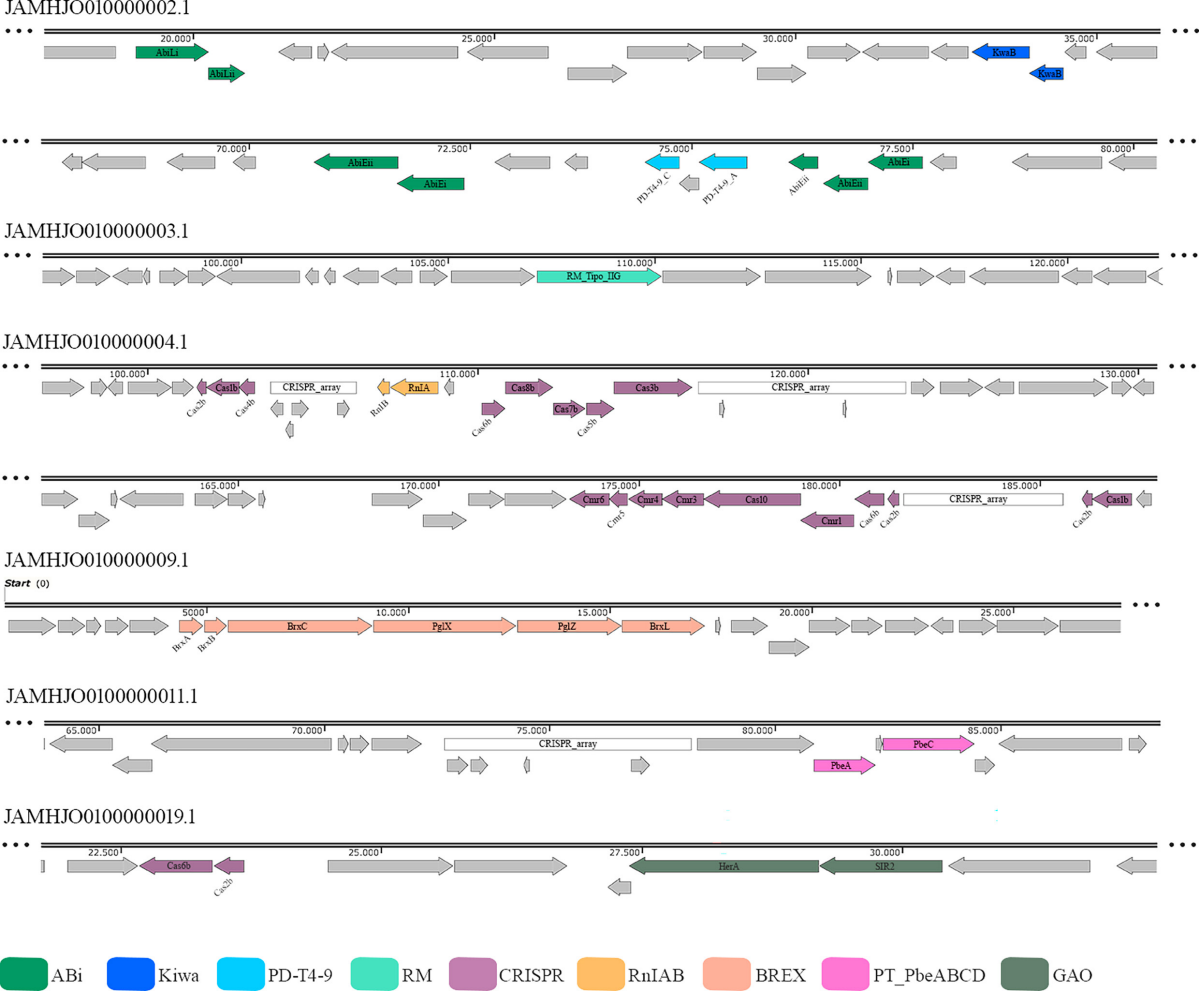
Schematic representation of gene clusters related to different antiphage systems. The genes were coloured according to the defence system, as indicated at the top.

Here, we analysed the presence of the different antiphage systems in the UFV_LIVM02 genome using the DefenseFinder online database and the PADLOC. The UFV_LIVM02 isolate harbours seven different antiphage systems as shown in Fig. 4. Three of them (Abi, PD-T4-9 and RnIAB) are based on the TA mechanism. It is known that most bacteria and archaea have TA systems, and these systems are improved through the acquisition of mobile genetic elements [76]. This system is based on the TA interaction, in which the toxins promote a reduction in metabolism and consequently limit the propagation of the phage and the antitoxins are RNA or proteins that neutralize the toxin or the RNA that encodes it [77]. The first is the Abi system, found in three distinct regions of the genome, two of them composed of the abiE operon and the third by the abiL operon, a system discovered in the 90s and widely studied [78,79]. The second, PD-T4-9, protects against infection by T4-type phages [80], and lastly, the RnIAB system. It is composed of the RnlA toxin that has RNase activity and the RnlB antitoxin that suppresses RNase activity [81].

In addition to the TA systems, other systems were identified in the UFV_LIVM02 genome. The Kiwa system was found, it is composed of two proteins KwaA and KwaB. KwaA detects phage infection by inhibiting the host’s RNA polymerase, which causes it to activate KwaB, which promotes a decrease in phage DNA replication through an exodeoxyribonuclease V-dependent pathway [82]. The RM system was also identified; this antiphage system recognizes invading DNA through the absence of methylation at the adenine or cytosine bases, cleaving it. Host DNA is normally methylated by methyltransferases after replication [83]. BREX and PT_PbeABCD were predicted, such systems allow phage adsorption but block phage DNA replication [84,85]. The GAO system is the sixth identified, and this system is made up of the HerA and Sir2 proteins, a system that induces bacterial death by depleting NAD ^+^ after phage infection [86].

In addition to the above antiphage systems, we also predicted the most popular antiphage system, CRISPR; in total, four CRISPR arrays were identified, with different numbers of spacers (Table 5). The *cas* genes identified in the UFV_LIMV02 strains are class I, types I and III and subtype I-B and III-B. CRISPR/Cas type I and type III systems share similar structures and likely evolved from a common ancestor [87]. However, each has a distinct mechanism for processing transcripts and destroying the target sequence; in the type I system, effector complexes recognize their DNA targets and then recruit the Cas3 protein to degrade the invading DNA, whereas, in type III, the Cas10 family proteins bind and cleave the ssRNA and are transcriptionally active DNA [88,89]. Furthermore, the UFV_LIMV02 strain showed a more significant number of spacers, indicating that this strain may have had greater exposure to different mobile genetic elements, such as bacteriophages, and plasmids made it possible to integrate these components into its genome as a defence mechanism.

**Table 5.** CRISPR arrays in the *O. teriensis* UFV_LIVM02

Contig	Start	End	Consensus_repeat	N_repeats
scaffold_4	103 722	106 320	TTTATATTCCAATATGGTTTGATTATTAT	40
scaffold_4	116 692	122 977	ATAATAATCAAACCATATTGGAATATAAAG	95
scaffold_4	181 608	185 586	GTCCTTATATATCCTTTATGGGTTAGAAAC	58
scaffold_11	72 682	78 146	GTCCTTATATATCCTTTATGGGTTAGAAAC	79

## Conclusion

The genome of the species *O. teriensis* strain UFV_LIMV02 shares large regions of synteny with the sequence of strain DSM 24906 deposited at NCBI. However, it is possible to observe some inversion parts. Analysis of AMR revealed that the isolate is resistant to six classes of antibiotics, being considered a multi-resistance bacteria. The *in silico* analysis revealed the presence of resistance genes with low similarity with ARGs deposited in databases, and being an indication of new ARGs not yet characterized, this would explain the phenotypic finding; in addition, the presence of five genomic islands contains genes related to resistance to heavy metals and antibiotics, several transposases and ISs. No prophages were found in its genome, but nine different defence systems against phage infection were detected. The results found in this study provide evidence that microorganisms found in offshore oil exploration residues may pose a risk for the spread of ARGs and other mobile genetic elements.

## supplementary material

10.1099/acmi.0.000801.v3 Uncited Supplementary Material 1.

## Abbreviations

ANI: average nucleic acid identity
ARGs: antibiotic resistance genes
CARD: comprehensive antibiotic resistance database
CRISPR: clustered regularly interspaced short palindromic repeats
dDDH: digital DNA–DNA hybridization
HGT: horizontal gene transfer
IS: insertion sequence
MIC: microbiologically induced corrosion
NCBI: National Center for Biotechnology Information
PADLOC: prokaryotic antiviral defence locator
RAST: rapid annotation using subsystem technology
SRB: sulfate-reducing bacteria
TRB: thiosulfate-reducing bacteria
