# Supplementary material for: Genomic analysis of Oceanotoga teriensis strain UFV_LIMV02, a multidrug-resistant thermophilic bacterium isolated from an offshore oil reservoir
Source: Access Microbiol. 2024 Aug 15;6(8):000801.v3. doi: 10.1099/acmi.0.000801.v3 (PMC11326445; doi:10.1099/acmi.0.000801.v3)
Supplement: Uncited Supplementary Material 1. [file acmi-6-00801-s001.pdf]

Genomic analysis of *Oceanotoga teriensis* strain UFV_LIMV02, a multidrug-resistant thermophilic bacterium isolated from an *offshore* oil reservoir

Adriele Jéssica do Carmo Santos[1], Roberto Sousa Dias[2], Carlos Henrique Martins da Silva[1], Pedro Marcus Pereira Vidigal[3], Maira Paula de Sousa[4], Cynthia Canedo da Silva[1], Sérgio Oliveira de Paula[2].

[1]Department of Microbiology, Federal University of Viçosa, Av. Peter Henry Rolfs, s/n, Campus Universitário, 36570-900, Viçosa, Minas Gerais, Brazil.

[2]Department of General Biology, Federal University of Viçosa, Av. Peter Henry Rolfs, s/n, Campus Universitário, 36570-900, Viçosa, Minas Gerais, Brazil.

[3]Center for Biomolecules Analysis (NuBIOMOL), Federal University of Viçosa, Vila Gianetti. Campus Universitário, 36570-900, Viçosa, Minas Gerais, Brazil.

[4]Leopoldo Américo Miguez de Mello Research and Development Center, Petrobras, Av. Horácio Macedo, 950, Federal University of Rio de Janeiro, 21941-915, Rio de Janeiro – Rio de Janeiro, Brazil

*Corresponding author: Departamento de Biologia Geral, Laboratório de Imunovirologia Molecular, Universidade Federal de Viçosa. Av.P.H.Rolfs, s/n, Campus Universitário, Viçosa-MG 36570-000
Email addresses: depaula@ufv.br (de Paula S.O)

Table S1. The composition of modified Postgate C medium.

**Composition**	**Value (g/L)**
NaCl	25.0
KH_2PO_4	0.5
NH_4Cl	1.0
Na_2SO_4	4.5
$CaCl_2.2H_2O$	0.04
$MgSO_4 \cdot 7H_2O$	0.064
Yeast extract	1.0
$C_6H_5Na_3O_7.2H_2O$	0.3
Glucose	0.25
$Na_2S_2O_3.5H_2O$	2.5
Resazurin	0.0001
Sodium thioglycolate	0.13

Table S2: Chemical classes, subclasses, and antibiotics used in this work.

Class	Subclass	Antibiotic*

Beta-lactam	Cephalosporin	Cefepime (CPM 30)
		Ceftriaxone (CRO 30)
		Ceftazidime (CAZ 30)
		Cefoxitin (CFO 30)
	Penicillin	Amoxicillin/Clavulanic Acid (AMC 30-20/10)
		Ampicillin (AMP 10)
		Piperacillin/Tazobactam (PIT 110)
	Monobactam	Aztreonam (ATM 30)
Macrolides	-	Azithromycin (AZI 15)
		Erythromycin (ERI 15)
Aminoglycosides	-	Gentamycin (GEN 10)
		Amikacin (AMI 30)
Lincosamides	-	Clindamycin (CLI 02)
Amphenicols	-	Chloramphenicol (CLO 30)
Tetracyclines	-	Tetracycline (TET 30)
Fluoroquinolones	-	Ciprofloxacin (CIP 05)
Glycopeptides	-	Vancomycin (VAN 30)
Oxazolidinones	-	Linezolid (LNZ 30)
Sulfonamides	-	Sulfamethoxazole/trimethoprim (SUT 25)
Rifamycin	-	Rifampin (RIF 05)

*Between parentheses are shown the code and antibiotic quantities in µg.

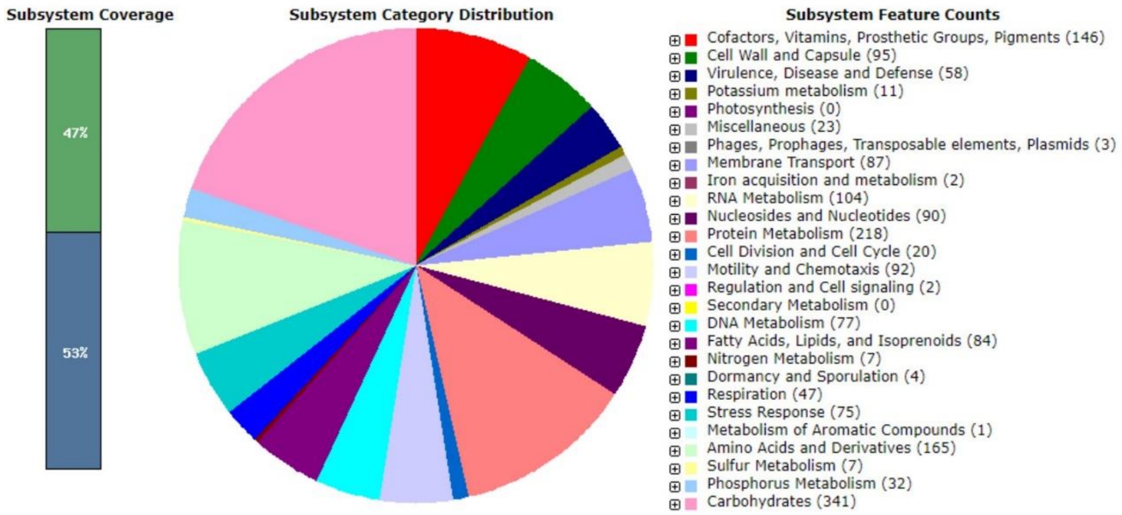

Supplementary Figure S1. The genome of *Oceanotoga teriensis* UFV_LIVM02 annotated by the Rapid Annotation System Technology (RAST) server, classified into subsystems and categories. The green bar on the left corresponds to the percentage of proteins annotated by RAST, while the bar indicates the percentage of proteins that cannot be annotated. The pie chart

demonstrates the abundance of each subsystem category and the count of each subsystem resource is shown in the right panel.

Table Supplementar S3: In silico detection of antibiotic resistance genes in *Oceanotoga teriensis* UFV_LIMV02 using the Comprehensive Antibiotic Resistance Database (CARD)

RGI Criteria	ARO Term	SNP	Detection Criteria	AMR Gene Family	Drug Class	Resistance Mechanism	% Identity of Matching Region	% Length od Refence Sequence
Loose	AAC(6')-Isa		protein homolog model	AAC(6')	aminoglycoside antibiotic	antibiotic inactivation	25.14	115.29
Loose	aadK		protein homolog model	ANT(6)	aminoglycoside antibiotic	antibiotic inactivation	37.94	102.11
Loose	AcrS		protein homolog model	resistance-nodulation-cell division (RND) antibiotic efflux pump	fluoroquinolone antibiotic, cephalosporin, glycylcycline, cephamycin, penam, tetracycline antibiotic, rifamycin antibiotic, phenicol antibiotic, disinfecting agents and antiseptics	antibiotic efflux	21.26	90.00
Loose	adeL		protein homolog model	resistance-nodulation-cell division (RND) antibiotic efflux pump	fluoroquinolone antibiotic, tetracycline antibiotic	antibiotic efflux	23.01	86.65
Loose	adeN		protein homolog model	resistance-nodulation-cell division (RND) antibiotic efflux pump	macrolide antibiotic, fluoroquinolone antibiotic, lincosamide antibiotic, carbapenem, cephalosporin, tetracycline antibiotic, rifamycin antibiotic, diaminopyrimidine antibiotic, phenicol antibiotic, penem	antibiotic efflux	31.36	91.71
Loose	adeR		protein homolog model	resistance-nodulation-cell division (RND) antibiotic efflux pump	glycylcycline, tetracycline antibiotic	antibiotic efflux	34.58	140.08
Loose	adeR		protein homolog model	resistance-nodulation-cell division (RND) antibiotic efflux pump	glycylcycline, tetracycline antibiotic	antibiotic efflux	24.44	95.14
Loose	Agrobacterium fabrum chloramphenicol acetyltransferase		protein homolog model	chloramphenicol acetyltransferase (CAT)	phenicol antibiotic	antibiotic inactivation	43.14	107.18
Loose	APH(2")-IIIa		protein homolog model	APH(2")	aminoglycoside antibiotic	antibiotic inactivation	25.23	89.22
Loose	apmA		protein homolog model	amp acetyltransferase	aminoglycoside antibiotic	antibiotic inactivation	39.44	73.36
Loose	arlR		protein homolog model	major facilitator superfamily (MFS) antibiotic efflux pump	fluoroquinolone antibiotic, disinfecting agents and antiseptics	antibiotic efflux	47.06	101.37
Loose	arlR		protein homolog model	major facilitator superfamily (MFS) antibiotic efflux pump	fluoroquinolone antibiotic, disinfecting agents and antiseptics	antibiotic efflux	33.18	102.28
Loose	arlR		protein homolog model	major facilitator superfamily (MFS) antibiotic efflux pump	fluoroquinolone antibiotic, disinfecting agents and antiseptics	antibiotic efflux	30.0	53.88
Loose	arlS		protein homolog model	major facilitator superfamily (MFS) antibiotic efflux pump	fluoroquinolone antibiotic, disinfecting agents and antiseptics	antibiotic efflux	34.04	95.12
Loose	arlS		protein homolog model	major facilitator superfamily (MFS) antibiotic efflux pump	fluoroquinolone antibiotic, disinfecting agents and antiseptics	antibiotic efflux	30.21	40.80
Loose	arlS		protein homolog model	major facilitator superfamily (MFS) antibiotic efflux pump	fluoroquinolone antibiotic, disinfecting agents and antiseptics	antibiotic efflux	28.89	70.29
Loose	arlS		protein homolog model	major facilitator superfamily (MFS) antibiotic efflux pump	fluoroquinolone antibiotic, disinfecting agents and antiseptics	antibiotic efflux	28.7	98.89
Loose	arlS		protein homolog model	major facilitator superfamily (MFS) antibiotic efflux pump	fluoroquinolone antibiotic, disinfecting agents and antiseptics	antibiotic efflux	27.31	229.49
Loose	arlS		protein homolog model	major facilitator superfamily (MFS) antibiotic efflux pump	fluoroquinolone antibiotic, disinfecting agents and antiseptics	antibiotic efflux	26.73	74.94
Loose	arlS		protein homolog model	major facilitator superfamily (MFS) antibiotic efflux pump	fluoroquinolone antibiotic, disinfecting agents and antiseptics	antibiotic efflux	25.81	104.21
Loose	arlS		protein homolog model	major facilitator superfamily (MFS) antibiotic efflux pump	fluoroquinolone antibiotic, disinfecting agents and antiseptics	antibiotic efflux	24.55	98.67
Loose	arnA		protein homolog model	pmr phosphoethanolamine transferase	peptide antibiotic	antibiotic target alteration	33.33	48.79
Loose	arnA		protein homolog model	pmr phosphoethanolamine transferase	peptide antibiotic	antibiotic target alteration	25.95	49.09
Loose	arnA		protein homolog model	pmr phosphoethanolamine transferase	peptide antibiotic	antibiotic target alteration	25.6	47.13
Loose	bacA		protein homolog model	undecaprenyl pyrophosphate related proteins	peptide antibiotic	antibiotic target alteration	28.79	92.67

Loose	BcII		protein homolog model	Bc beta-lactamase	cephalosporin, penam	antibiotic inactivation	25.32	73.05
Loose	bcrA		protein homolog model	ATP-binding cassette (ABC) antibiotic efflux pump	peptide antibiotic	antibiotic efflux	38.43	81.37
Loose	bcrA		protein homolog model	ATP-binding cassette (ABC) antibiotic efflux pump	peptide antibiotic	antibiotic efflux	38.43	81.37
Loose	bcrA		protein homolog model	ATP-binding cassette (ABC) antibiotic efflux pump	peptide antibiotic	antibiotic efflux	36.13	93.46
Loose	bcrA		protein homolog model	ATP-binding cassette (ABC) antibiotic efflux pump	peptide antibiotic	antibiotic efflux	34.5	82.35
Loose	bcrA		protein homolog model	ATP-binding cassette (ABC) antibiotic efflux pump	peptide antibiotic	antibiotic efflux	34.39	98.04
Loose	bcrA		protein homolog model	ATP-binding cassette (ABC) antibiotic efflux pump	peptide antibiotic	antibiotic efflux	34.12	96.73
Loose	bcrA		protein homolog model	ATP-binding cassette (ABC) antibiotic efflux pump	peptide antibiotic	antibiotic efflux	33.33	172.22
Loose	bcrA		protein homolog model	ATP-binding cassette (ABC) antibiotic efflux pump	peptide antibiotic	antibiotic efflux	31.78	77.78
Loose	bcrA		protein homolog model	ATP-binding cassette (ABC) antibiotic efflux pump	peptide antibiotic	antibiotic efflux	31.28	76.14
Loose	bcrA		protein homolog model	ATP-binding cassette (ABC) antibiotic efflux pump	peptide antibiotic	antibiotic efflux	31.12	100.33
Loose	bcrA		protein homolog model	ATP-binding cassette (ABC) antibiotic efflux pump	peptide antibiotic	antibiotic efflux	31.08	92.81
Loose	bcrA		protein homolog model	ATP-binding cassette (ABC) antibiotic efflux pump	peptide antibiotic	antibiotic efflux	30.87	76.80
Loose	bcrA		protein homolog model	ATP-binding cassette (ABC) antibiotic efflux pump	peptide antibiotic	antibiotic efflux	30.65	87.91
Loose	bcrA		protein homolog model	ATP-binding cassette (ABC) antibiotic efflux pump	peptide antibiotic	antibiotic efflux	30.09	85.62
Loose	bcrA		protein homolog model	ATP-binding cassette (ABC) antibiotic efflux pump	peptide antibiotic	antibiotic efflux	28.57	83.01
Loose	bcrA		protein homolog model	ATP-binding cassette (ABC) antibiotic efflux pump	peptide antibiotic	antibiotic efflux	26.34	97.71
Loose	bcrA		protein homolog model	ATP-binding cassette (ABC) antibiotic efflux pump	peptide antibiotic	antibiotic efflux	26.27	162.75
Loose	bcrA		protein homolog model	ATP-binding cassette (ABC) antibiotic efflux pump	peptide antibiotic	antibiotic efflux	25.67	92.48
Loose	Borreliella burgdorferi murA with mutation conferring resistance to fosfomycin	D116C	protein variant model	antibiotic-resistant murA transferase	Fosfomycin	antibiotic target alteration	35.61	100.70
Loose	catB8		protein homolog model	chloramphenicol acetyltransferase (CAT)	phenicol antibiotic	antibiotic inactivation	47.69	89.52
Loose	cdeA		protein homolog model	multidrug and toxic compound extrusion (MATE) transporter	fluoroquinolone antibiotic, disinfecting agents and antiseptics	antibiotic efflux	40.32	102.49
Loose	cdeA		protein homolog model	multidrug and toxic compound extrusion (MATE) transporter	fluoroquinolone antibiotic, disinfecting agents and antiseptics	antibiotic efflux	35.38	99.55
Loose	cdeA		protein homolog model	multidrug and toxic compound extrusion (MATE) transporter	fluoroquinolone antibiotic, disinfecting agents and antiseptics	antibiotic efflux	34.19	100.45
Loose	cdeA		protein homolog model	multidrug and toxic compound extrusion (MATE) transporter	fluoroquinolone antibiotic, disinfecting agents and antiseptics	antibiotic efflux	31.28	100.00
Loose	cdeA		protein homolog model	multidrug and toxic compound extrusion (MATE) transporter	fluoroquinolone antibiotic, disinfecting agents and antiseptics	antibiotic efflux	30.64	100.45
Loose	cdeA		protein homolog model	multidrug and toxic compound extrusion (MATE) transporter	fluoroquinolone antibiotic, disinfecting agents and antiseptics	antibiotic efflux	26.32	106.58
Loose	cdeA		protein homolog model	multidrug and toxic compound extrusion (MATE) transporter	fluoroquinolone antibiotic, disinfecting agents and antiseptics	antibiotic efflux	26.21	99.32
Loose	cdeA		protein homolog model	multidrug and toxic compound extrusion (MATE) transporter	fluoroquinolone antibiotic, disinfecting agents and antiseptics	antibiotic efflux	26.03	102.04
Loose	cdeA		protein homolog model	multidrug and toxic compound extrusion (MATE) transporter	fluoroquinolone antibiotic, disinfecting agents and antiseptics	antibiotic efflux	23.47	101.36
Loose	cfr(D)		protein homolog model	Cfr 23S ribosomal RNA methyltransferase	lincosamide antibiotic, streptogramin antibiotic, oxazolidinone antibiotic, phenicol antibiotic	antibiotic target alteration	18.4	86.27

Loose	clbA		protein homolog model	Cfr 23S ribosomal RNA methyltransferase	lincosamide antibiotic, streptogramin antibiotic, streptogramin A antibiotic, oxazolidinone antibiotic, phenicol antibiotic, pleuromutilin antibiotic	antibiotic target alteration	34.95	97.99
Loose	Clostridioides difficile gyrA conferring resistance to fluoroquinolones	P116A	protein variant model	fluoroquinolone resistant gyrA	fluoroquinolone antibiotic	antibiotic target alteration	44.65	102.10
Loose	Clostridioides difficile gyrB conferring resistance to fluoroquinolones	V423F	protein variant model	fluoroquinolone resistant gyrB	fluoroquinolone antibiotic	antibiotic target alteration	51.79	100.63
Loose	cmeA		protein homolog model	resistance-nodulation-cell division (RND) antibiotic efflux pump	macrolide antibiotic, fluoroquinolone antibiotic, cephalosporin, fusidic acid	antibiotic efflux	25.11	88.28
Loose	cmeR		protein homolog model	resistance-nodulation-cell division (RND) antibiotic efflux pump	macrolide antibiotic, fluoroquinolone antibiotic, cephalosporin, fusidic acid	antibiotic efflux	39.29	97.14
Loose	cmeR		protein homolog model	resistance-nodulation-cell division (RND) antibiotic efflux pump	macrolide antibiotic, fluoroquinolone antibiotic, cephalosporin, fusidic acid	antibiotic efflux	27.19	98.10
Loose	cpxA		protein homolog model	resistance-nodulation-cell division (RND) antibiotic efflux pump	aminoglycoside antibiotic, aminocoumarin antibiotic	antibiotic efflux	28.43	29.32
Loose	cpxA		protein homolog model	resistance-nodulation-cell division (RND) antibiotic efflux pump	aminoglycoside antibiotic, aminocoumarin antibiotic	antibiotic efflux	26.09	86.65
Loose	CRD3-1		protein homolog model	CRD3 beta-lactamase	carbapenem	antibiotic inactivation	41.27	77.56
Loose	CRP		protein homolog model	resistance-nodulation-cell division (RND) antibiotic efflux pump	macrolide antibiotic, fluoroquinolone antibiotic, penam	antibiotic efflux	22.33	110.48
Loose	EdeQ		protein homolog model	Edeine acetyltransferase	peptide antibiotic, polyamine antibiotic	antibiotic inactivation	38.13	97.24
Loose	efrA		protein homolog model	ATP-binding cassette (ABC) antibiotic efflux pump	macrolide antibiotic, fluoroquinolone antibiotic, rifamycin antibiotic	antibiotic efflux	46.86	128.00
Loose	Erm(48)		protein homolog model	Erm 23S ribosomal RNA methyltransferase	macrolide antibiotic, lincosamide antibiotic, streptogramin antibiotic	antibiotic target alteration	28.77	109.05
Loose	ErmD		protein homolog model	Erm 23S ribosomal RNA methyltransferase	macrolide antibiotic, lincosamide antibiotic, streptogramin antibiotic, streptogramin A antibiotic, streptogramin B antibiotic	antibiotic target alteration	25.53	87.46
Loose	ErmE		protein homolog model	Erm 23S ribosomal RNA methyltransferase	macrolide antibiotic, lincosamide antibiotic, streptogramin antibiotic, streptogramin A antibiotic, streptogramin B antibiotic	antibiotic target alteration	26.07	69.03
Loose	Escherichia coli AcrAB-TolC with AcrR mutation conferring resistance to ciprofloxacin, tetracycline, and ceftazidime		protein overexpression model	resistance-nodulation-cell division (RND) antibiotic efflux pump	fluoroquinolone antibiotic, cephalosporin, glycylcycline, penam, tetracycline antibiotic, rifamycin antibiotic, phenicol antibiotic, disinfecting agents and antiseptics	antibiotic target alteration, antibiotic efflux	38.24	101.40
Loose	Escherichia coli AcrAB-TolC with AcrR mutation conferring resistance to ciprofloxacin, tetracycline, and ceftazidime		protein overexpression model	resistance-nodulation-cell division (RND) antibiotic efflux pump	fluoroquinolone antibiotic, cephalosporin, glycylcycline, penam, tetracycline antibiotic, rifamycin antibiotic, phenicol antibiotic, disinfecting agents and antiseptics	antibiotic target alteration, antibiotic efflux	30.0	174.42
Loose	Escherichia coli AcrAB-TolC with AcrR mutation conferring resistance to ciprofloxacin,		protein overexpression model	resistance-nodulation-cell division (RND) antibiotic efflux pump	fluoroquinolone antibiotic, cephalosporin, glycylcycline, penam, tetracycline antibiotic, rifamycin antibiotic, phenicol antibiotic, disinfecting agents and antiseptics	antibiotic target alteration, antibiotic efflux	30.0	89.30

	tetracycline, and ceftazidime							
Loose	Escherichia coli ampC1 beta-lactamase		protein homolog model	ampC-type beta-lactamase	cephalosporin, penam	antibiotic inactivation	36.71	123.04
Loose	Escherichia coli ampC1 beta-lactamase		protein homolog model	ampC-type beta-lactamase	cephalosporin, penam	antibiotic inactivation	26.32	76.73
Loose	Escherichia coli EF-Tu mutants conferring resistance to Pulvomycin	R234F	protein variant model	elfamycin resistant EF-Tu	elfamycin antibiotic	antibiotic target alteration	68.34	97.56
Loose	Escherichia coli PtsI with mutation conferring resistance to fosfomycin	V25I	protein variant model	antibiotic-resistant ptsI phosphotransferase	fosfomycin	antibiotic target alteration	43.72	98.26
Loose	Escherichia coli soxS with mutation conferring antibiotic resistance		protein overexpression model	ATP-binding cassette (ABC) antibiotic efflux pump, major facilitator superfamily (MFS) antibiotic efflux pump, resistance-nodulation-cell division (RND) antibiotic efflux pump, General Bacterial Porin with reduced permeability to beta-lactams	fluoroquinolone antibiotic, monobactam, carbapenem, cephalosporin, glycylcycline, cephamycin, penam, tetracycline antibiotic, rifamycin antibiotic, phenicol antibiotic, penem, disinfecting agents and antiseptics	antibiotic target alteration, antibiotic efflux, reduced permeability to antibiotic	31.63	295.33
Loose	Escherichia coli soxS with mutation conferring antibiotic resistance		protein overexpression model	ATP-binding cassette (ABC) antibiotic efflux pump, major facilitator superfamily (MFS) antibiotic efflux pump, resistance-nodulation-cell division (RND) antibiotic efflux pump, General Bacterial Porin with reduced permeability to beta-lactams	fluoroquinolone antibiotic, monobactam, carbapenem, cephalosporin, glycylcycline, cephamycin, penam, tetracycline antibiotic, rifamycin antibiotic, phenicol antibiotic, penem, disinfecting agents and antiseptics	antibiotic target alteration, antibiotic efflux, reduced permeability to antibiotic	27.72	235.51
Loose	Escherichia coli soxS with mutation conferring antibiotic resistance		protein overexpression model	ATP-binding cassette (ABC) antibiotic efflux pump, major facilitator superfamily (MFS) antibiotic efflux pump, resistance-nodulation-cell division (RND) antibiotic efflux pump, General Bacterial Porin with reduced permeability to beta-lactams	fluoroquinolone antibiotic, monobactam, carbapenem, cephalosporin, glycylcycline, cephamycin, penam, tetracycline antibiotic, rifamycin antibiotic, phenicol antibiotic, penem, disinfecting agents and antiseptics	antibiotic target alteration, antibiotic efflux, reduced permeability to antibiotic	25.74	265.42
Loose	evgA		protein homolog model	major facilitator superfamily (MFS) antibiotic efflux pump, resistance-nodulation-cell division (RND) antibiotic efflux pump	macrolide antibiotic, fluoroquinolone antibiotic, penam, tetracycline antibiotic	antibiotic efflux	26.17	107.35
Loose	evgA		protein homolog model	major facilitator superfamily (MFS) antibiotic efflux pump, resistance-nodulation-cell division (RND) antibiotic efflux pump	macrolide antibiotic, fluoroquinolone antibiotic, penam, tetracycline antibiotic	antibiotic efflux	23.91	93.63
Loose	evgS		protein homolog model	major facilitator superfamily (MFS) antibiotic efflux pump, resistance-nodulation-cell division (RND) antibiotic efflux pump	macrolide antibiotic, fluoroquinolone antibiotic, penam, tetracycline antibiotic	antibiotic efflux	29.92	53.80
Loose	evgS		protein homolog model	major facilitator superfamily (MFS) antibiotic efflux pump, resistance-nodulation-cell division (RND) antibiotic efflux pump	macrolide antibiotic, fluoroquinolone antibiotic, penam, tetracycline antibiotic	antibiotic efflux	28.84	74.52
Loose	evgS		protein homolog model	major facilitator superfamily (MFS) antibiotic efflux pump, resistance-nodulation-cell division (RND) antibiotic efflux pump	macrolide antibiotic, fluoroquinolone antibiotic, penam, tetracycline antibiotic	antibiotic efflux	27.08	41.77
Loose	evgS		protein homolog model	major facilitator superfamily (MFS) antibiotic efflux pump, resistance-nodulation-cell division (RND) antibiotic efflux pump	macrolide antibiotic, fluoroquinolone antibiotic, penam, tetracycline antibiotic	antibiotic efflux	26.24	55.56
Loose	GOB-46		protein homolog model	GOB beta-lactamase	carbapenem, cephalosporin, penam	antibiotic inactivation	27.67	88.62
Loose	golS		protein homolog model	resistance-nodulation-cell division (RND) antibiotic efflux pump	monobactam, carbapenem, cephalosporin, cephamycin, penam, phenicol antibiotic, penem	antibiotic efflux	28.97	184.42
Loose	golS		protein homolog model	resistance-nodulation-cell division (RND) antibiotic efflux pump	monobactam, carbapenem, cephalosporin, cephamycin, penam, phenicol antibiotic, penem	antibiotic efflux	24.27	95.45

Loose	golS		protein homolog model	resistance-nodulation-cell division (RND) antibiotic efflux pump	monobactam, carbapenem, cephalosporin, cephamycin, penam, phenicol antibiotic, penem	antibiotic efflux	23.85	161.69
Loose	Helicobacter pylori pbp1 mutants conferring resistance to amoxicillin	S414N, T593S	protein variant model	Penicillin-binding protein mutations conferring resistance to beta-lactam antibiotics	cephalosporin, cephamycin, penam	antibiotic target alteration	28.69	105.77
Loose	hmrM		protein homolog model	multidrug and toxic compound extrusion (MATE) transporter	fluoroquinolone antibiotic, disinfecting agents and antiseptics	antibiotic efflux	27.36	97.41
Loose	hp1165		protein homolog model	major facilitator superfamily (MFS) antibiotic efflux pump	tetracycline antibiotic	antibiotic efflux	24.8	101.55
Loose	IMP-25		protein homolog model	IMP beta-lactamase	carbapenem, cephalosporin, cephamycin, penam, penem	antibiotic inactivation	25.15	97.97
Loose	kdpE		protein homolog model	kdpDE	aminoglycoside antibiotic	antibiotic efflux	36.11	154.67
Loose	kdpE		protein homolog model	kdpDE	aminoglycoside antibiotic	antibiotic efflux	25.23	60.89
Loose	LlmA 23S ribosomal RNA methyltransferase		protein homolog model	Llm 23S ribosomal RNA methyltransferase	lincosamide antibiotic	antibiotic target alteration	28.91	134.15
Loose	lmrD		protein homolog model	ATP-binding cassette (ABC) antibiotic efflux pump	lincosamide antibiotic	antibiotic efflux	31.93	80.87
Loose	LpsB		protein homolog model	Intrinsic peptide antibiotic resistant Lps	peptide antibiotic	reduced permeability to antibiotic	24.22	104.64
Loose	lsaC		protein homolog model	lsa-type ABC-F protein	lincosamide antibiotic, streptogramin antibiotic, pleuromutilin antibiotic	antibiotic target protection	38.08	104.47
Loose	lsaC		protein homolog model	lsa-type ABC-F protein	lincosamide antibiotic, streptogramin antibiotic, pleuromutilin antibiotic	antibiotic target protection	31.91	52.24
Loose	lsaC		protein homolog model	lsa-type ABC-F protein	lincosamide antibiotic, streptogramin antibiotic, pleuromutilin antibiotic	antibiotic target protection	26.46	49.39
Loose	lsaC		protein homolog model	lsa-type ABC-F protein	lincosamide antibiotic, streptogramin antibiotic, pleuromutilin antibiotic	antibiotic target protection	23.43	115.04
Loose	macB		protein homolog model	ATP-binding cassette (ABC) antibiotic efflux pump	macrolide antibiotic	antibiotic efflux	46.93	35.87
Loose	macB		protein homolog model	ATP-binding cassette (ABC) antibiotic efflux pump	macrolide antibiotic	antibiotic efflux	38.84	35.56
Loose	macB		protein homolog model	ATP-binding cassette (ABC) antibiotic efflux pump	macrolide antibiotic	antibiotic efflux	38.03	37.42
Loose	macB		protein homolog model	ATP-binding cassette (ABC) antibiotic efflux pump	macrolide antibiotic	antibiotic efflux	37.56	34.94
Loose	macB		protein homolog model	ATP-binding cassette (ABC) antibiotic efflux pump	macrolide antibiotic	antibiotic efflux	37.39	39.44
Loose	macB		protein homolog model	ATP-binding cassette (ABC) antibiotic efflux pump	macrolide antibiotic	antibiotic efflux	34.4	63.51
Loose	macB		protein homolog model	ATP-binding cassette (ABC) antibiotic efflux pump	macrolide antibiotic	antibiotic efflux	34.38	38.82
Loose	macB		protein homolog model	ATP-binding cassette (ABC) antibiotic efflux pump	macrolide antibiotic	antibiotic efflux	32.98	145.81
Loose	macB		protein homolog model	ATP-binding cassette (ABC) antibiotic efflux pump	macrolide antibiotic	antibiotic efflux	31.87	50.00
Loose	macB		protein homolog model	ATP-binding cassette (ABC) antibiotic efflux pump	macrolide antibiotic	antibiotic efflux	30.58	48.91
Loose	macB		protein homolog model	ATP-binding cassette (ABC) antibiotic efflux pump	macrolide antibiotic	antibiotic efflux	30.51	49.53
Loose	macB		protein homolog model	ATP-binding cassette (ABC) antibiotic efflux pump	macrolide antibiotic	antibiotic efflux	30.42	54.04
Loose	macB		protein homolog model	ATP-binding cassette (ABC) antibiotic efflux pump	macrolide antibiotic	antibiotic efflux	29.86	40.99
Loose	macB		protein homolog model	ATP-binding cassette (ABC) antibiotic efflux pump	macrolide antibiotic	antibiotic efflux	29.22	31.68
Loose	macB		protein homolog model	ATP-binding cassette (ABC) antibiotic efflux pump	macrolide antibiotic	antibiotic efflux	29.13	77.33
Loose	macB		protein homolog model	ATP-binding cassette (ABC) antibiotic efflux pump	macrolide antibiotic	antibiotic efflux	27.47	50.62
Loose	macB		protein homolog model	ATP-binding cassette (ABC) antibiotic efflux pump	macrolide antibiotic	antibiotic efflux	27.08	178.26
Loose	macB		protein homolog model	ATP-binding cassette (ABC) antibiotic efflux pump	macrolide antibiotic	antibiotic efflux	26.89	51.09
Loose	macB		protein homolog model	ATP-binding cassette (ABC) antibiotic efflux pump	macrolide antibiotic	antibiotic efflux	25.5	52.64
Loose	marA		protein homolog model	resistance-nodulation-cell division (RND) antibiotic efflux pump, General Bacterial Porin with reduced permeability to beta-lactams	fluoroquinolone antibiotic, monobactam, carbapenem, cephalosporin, glycylcycline, cephamycin, penam, tetracycline antibiotic, rifamycin antibiotic, phenicol antibiotic, penem, disinfecting agents and antiseptics	antibiotic efflux, reduced permeability to antibiotic	37.5	221.26
Loose	marA		protein homolog model	resistance-nodulation-cell division (RND) antibiotic efflux pump, General Bacterial Porin with reduced permeability to beta-lactams	fluoroquinolone antibiotic, monobactam, carbapenem, cephalosporin, glycylcycline, cephamycin, penam, tetracycline antibiotic, rifamycin antibiotic, phenicol antibiotic, penem, disinfecting agents and antiseptics	antibiotic efflux, reduced permeability to antibiotic	29.13	255.91
Loose	marA		protein homolog model	resistance-nodulation-cell division (RND) antibiotic efflux pump, General Bacterial Porin with reduced permeability to beta-lactams	fluoroquinolone antibiotic, monobactam, carbapenem, cephalosporin, glycylcycline, cephamycin, penam, tetracycline antibiotic, rifamycin antibiotic, phenicol antibiotic, penem, disinfecting agents and antiseptics	antibiotic efflux, reduced permeability to antibiotic	26.32	251.18

Loose	mdtC		protein homolog model	resistance-nodulation-cell division (RND) antibiotic efflux pump	aminocoumarin antibiotic	antibiotic efflux	25.33	88.20
Loose	MdtK		protein homolog model	multidrug and toxic compound extrusion (MATE) transporter	fluoroquinolone antibiotic	antibiotic efflux	20.91	93.67
Loose	mecA		protein homolog model	methicillin resistant PBP2	penam	antibiotic target replacement	26.25	85.03
Loose	mefH		protein homolog model	major facilitator superfamily (MFS) antibiotic efflux pump	macrolide antibiotic	antibiotic efflux	39.25	369.72
Loose	mel		protein homolog model	msr-type ABC-F protein	macrolide antibiotic, streptogramin antibiotic	antibiotic target protection	23.82	103.21
Loose	mepA		protein homolog model	multidrug and toxic compound extrusion (MATE) transporter	glycylcycline, tetracycline antibiotic	antibiotic efflux	31.78	101.11
Loose	mepA		protein homolog model	multidrug and toxic compound extrusion (MATE) transporter	glycylcycline, tetracycline antibiotic	antibiotic efflux	27.68	100.00
Loose	mepA		protein homolog model	multidrug and toxic compound extrusion (MATE) transporter	glycylcycline, tetracycline antibiotic	antibiotic efflux	24.94	100.44
Loose	mepA		protein homolog model	multidrug and toxic compound extrusion (MATE) transporter	glycylcycline, tetracycline antibiotic	antibiotic efflux	23.74	101.33
Loose	mepA		protein homolog model	multidrug and toxic compound extrusion (MATE) transporter	glycylcycline, tetracycline antibiotic	antibiotic efflux	21.63	100.22
Loose	mepR		protein homolog model	multidrug and toxic compound extrusion (MATE) transporter	glycylcycline, tetracycline antibiotic	antibiotic efflux	23.01	105.76
Loose	mgrA		protein homolog model	ATP-binding cassette (ABC) antibiotic efflux pump, major facilitator superfamily (MFS) antibiotic efflux pump	fluoroquinolone antibiotic, cephalosporin, penam, tetracycline antibiotic, peptide antibiotic, disinfecting agents and antiseptics	antibiotic efflux	31.69	95.92
Loose	mgrA		protein homolog model	ATP-binding cassette (ABC) antibiotic efflux pump, major facilitator superfamily (MFS) antibiotic efflux pump	fluoroquinolone antibiotic, cephalosporin, penam, tetracycline antibiotic, peptide antibiotic, disinfecting agents and antiseptics	antibiotic efflux	29.03	99.32
Loose	mgrA		protein homolog model	ATP-binding cassette (ABC) antibiotic efflux pump, major facilitator superfamily (MFS) antibiotic efflux pump	fluoroquinolone antibiotic, cephalosporin, penam, tetracycline antibiotic, peptide antibiotic, disinfecting agents and antiseptics	antibiotic efflux	28.57	102.04
Loose	mgrA		protein homolog model	ATP-binding cassette (ABC) antibiotic efflux pump, major facilitator superfamily (MFS) antibiotic efflux pump	fluoroquinolone antibiotic, cephalosporin, penam, tetracycline antibiotic, peptide antibiotic, disinfecting agents and antiseptics	antibiotic efflux	26.23	100.68
Loose	mgrA		protein homolog model	ATP-binding cassette (ABC) antibiotic efflux pump, major facilitator superfamily (MFS) antibiotic efflux pump	fluoroquinolone antibiotic, cephalosporin, penam, tetracycline antibiotic, peptide antibiotic, disinfecting agents and antiseptics	antibiotic efflux	24.8	102.72
Loose	mreA		protein homolog model	major facilitator superfamily (MFS) antibiotic efflux pump	macrolide antibiotic	antibiotic efflux	31.06	94.86
Loose	msbA		protein homolog model	ATP-binding cassette (ABC) antibiotic efflux pump	nitroimidazole antibiotic	antibiotic efflux	32.09	99.48
Loose	msbA		protein homolog model	ATP-binding cassette (ABC) antibiotic efflux pump	nitroimidazole antibiotic	antibiotic efflux	30.84	98.63
Loose	msbA		protein homolog model	ATP-binding cassette (ABC) antibiotic efflux pump	nitroimidazole antibiotic	antibiotic efflux	30.04	101.03
Loose	msbA		protein homolog model	ATP-binding cassette (ABC) antibiotic efflux pump	nitroimidazole antibiotic	antibiotic efflux	29.37	100.52
Loose	msbA		protein homolog model	ATP-binding cassette (ABC) antibiotic efflux pump	nitroimidazole antibiotic	antibiotic efflux	29.18	102.41
Loose	msbA		protein homolog model	ATP-binding cassette (ABC) antibiotic efflux pump	nitroimidazole antibiotic	antibiotic efflux	29.06	100.34
Loose	msbA		protein homolog model	ATP-binding cassette (ABC) antibiotic efflux pump	nitroimidazole antibiotic	antibiotic efflux	28.63	45.19
Loose	msbA		protein homolog model	ATP-binding cassette (ABC) antibiotic efflux pump	nitroimidazole antibiotic	antibiotic efflux	28.57	55.67
Loose	msbA		protein homolog model	ATP-binding cassette (ABC) antibiotic efflux pump	nitroimidazole antibiotic	antibiotic efflux	27.52	44.50
Loose	msbA		protein homolog model	ATP-binding cassette (ABC) antibiotic efflux pump	nitroimidazole antibiotic	antibiotic efflux	27.27	44.16
Loose	msbA		protein homolog model	ATP-binding cassette (ABC) antibiotic efflux pump	nitroimidazole antibiotic	antibiotic efflux	21.34	42.96
Loose	mtrA		protein homolog model	resistance-nodulation-cell division (RND) antibiotic efflux pump	macrolide antibiotic, penam	antibiotic efflux	44.87	106.14
Loose	mtrA		protein homolog model	resistance-nodulation-cell division (RND) antibiotic efflux pump	macrolide antibiotic, penam	antibiotic efflux	36.53	96.49
Loose	mtrA		protein homolog model	resistance-nodulation-cell division (RND) antibiotic efflux pump	macrolide antibiotic, penam	antibiotic efflux	29.66	53.07
Loose	Mycobacterium tuberculosis kasA mutant conferring	D66N	protein variant model	antibiotic resistant kasA	isoniazid	antibiotic target alteration	32.76	98.80

	resistance to isoniazid							
Loose	Mycobacterium tuberculosis pncA mutations conferring resistance to pyrazinamide	T47S, W119R	protein variant model	Pyrazinamide resistant pncA	Pyrazinamide]	antibiotic target alteration	28.49	104.30
Loose	Mycobacterium tuberculosis pncA mutations conferring resistance to pyrazinamide	D136N, H59D, G97S	protein variant model	Pyrazinamide resistant pncA	pyrazinamide	antibiotic target alteration	25.68	101.08
Loose	Mycobacterium tuberculosis rpoB mutants conferring resistance to rifampicin	H526S, I572F, D516G, S512R, T508A	protein variant model	rifamycin-resistant beta-subunit of RNA polymerase (rpoB)	rifamycin antibiotic	antibiotic target alteration, antibiotic target replacement	48.11	101.28
Loose	Mycolicibacterium smegmatis ndh with mutation conferring resistance to isoniazid	G84D, Q335H	protein variant model	antibiotic resistant ndh	isoniazid	antibiotic target alteration	31.55	137.20
Loose	nalD		protein overexpression model	resistance-nodulation-cell division (RND) antibiotic efflux pump	macrolide antibiotic, fluoroquinolone antibiotic, monobactam, carbapenem, cephalosporin, cephamycin, penam, tetracycline antibiotic, peptide antibiotic, aminocoumarin antibiotic, diaminopyrimidine antibiotic, sulfonamide antibiotic, phenicol antibiotic, penem	antibiotic efflux	30.26	114.15
Loose	nalD		protein overexpression model	resistance-nodulation-cell division (RND) antibiotic efflux pump	macrolide antibiotic, fluoroquinolone antibiotic, monobactam, carbapenem, cephalosporin, cephamycin, penam, tetracycline antibiotic, peptide antibiotic, aminocoumarin antibiotic, diaminopyrimidine antibiotic, sulfonamide antibiotic, phenicol antibiotic, penem	antibiotic efflux	25.42	175.94
Loose	norB		protein homolog model	major facilitator superfamily (MFS) antibiotic efflux pump	fluoroquinolone antibiotic	antibiotic efflux	24.86	85.19
Loose	novA		protein homolog model	ATP-binding cassette (ABC) antibiotic efflux pump	aminocoumarin antibiotic	antibiotic efflux	40.87	97.24
Loose	novA		protein homolog model	ATP-binding cassette (ABC) antibiotic efflux pump	aminocoumarin antibiotic	antibiotic efflux	36.04	63.58
Loose	novA		protein homolog model	ATP-binding cassette (ABC) antibiotic efflux pump	aminocoumarin antibiotic	antibiotic efflux	32.47	93.50
Loose	optrA		protein homolog model	Miscellaneous ABC-F subfamily ATP-binding cassette ribosomal protection proteins	oxazolidinone antibiotic, phenicol antibiotic	antibiotic target protection	29.59	91.45
Loose	optrA		protein homolog model	Miscellaneous ABC-F subfamily ATP-binding cassette ribosomal protection proteins	oxazolidinone antibiotic, phenicol antibiotic	antibiotic target protection	24.16	71.76
Loose	patA		protein homolog model	ATP-binding cassette (ABC) antibiotic efflux pump	fluoroquinolone antibiotic	antibiotic efflux	28.15	45.04
Loose	patA		protein homolog model	ATP-binding cassette (ABC) antibiotic efflux pump	fluoroquinolone antibiotic	antibiotic efflux	27.95	43.97
Loose	patA		protein homolog model	ATP-binding cassette (ABC) antibiotic efflux pump	fluoroquinolone antibiotic	antibiotic efflux	27.52	43.97
Loose	patB		protein homolog model	ATP-binding cassette (ABC) antibiotic efflux pump	fluoroquinolone antibiotic	antibiotic efflux	35.94	38.61
Loose	patB		protein homolog model	ATP-binding cassette (ABC) antibiotic efflux pump	fluoroquinolone antibiotic	antibiotic efflux	34.69	104.93
Loose	patB		protein homolog model	ATP-binding cassette (ABC) antibiotic efflux pump	fluoroquinolone antibiotic	antibiotic efflux	31.05	109.52
Loose	patB		protein homolog model	ATP-binding cassette (ABC) antibiotic efflux pump	fluoroquinolone antibiotic	antibiotic efflux	30.19	98.64
Loose	patB		protein homolog model	ATP-binding cassette (ABC) antibiotic efflux pump	fluoroquinolone antibiotic	antibiotic efflux	28.69	78.23
Loose	patB		protein homolog model	ATP-binding cassette (ABC) antibiotic efflux pump	fluoroquinolone antibiotic	antibiotic efflux	23.85	95.24
Loose	PDC-103		protein homolog model	PDC beta-lactamase	monobactam, carbapenem, cephalosporin	antibiotic inactivation	36.23	23.43
Loose	PmrF		protein homolog model	pmr phosphoethanolamine transferase	peptide antibiotic	antibiotic target alteration	24.85	109.94
Loose	POM-1		protein homolog model	POM beta-lactamase	carbapenem	antibiotic inactivation	38.3	79.37
Loose	poxtA		protein homolog model	Miscellaneous ABC-F subfamily ATP-binding cassette ribosomal protection proteins	tetracycline antibiotic, oxazolidinone antibiotic, phenicol antibiotic	antibiotic target protection	33.48	45.57

Loose	poxtA		protein homolog model	Miscellaneous ABC-F subfamily ATP-binding cassette ribosomal protection proteins	tetracycline antibiotic, oxazolidinone antibiotic, phenicol antibiotic	antibiotic target protection	24.92	72.14
Loose	Pseudomonas aeruginosa catB6		protein homolog model	chloramphenicol acetyltransferase (CAT)	phenicol antibiotic	antibiotic inactivation	60.91	99.05
Loose	Pseudomonas aeruginosa soxR		protein homolog model	ATP-binding cassette (ABC) antibiotic efflux pump, major facilitator superfamily (MFS) antibiotic efflux pump, resistance-nodulation-cell division (RND) antibiotic efflux pump	fluoroquinolone antibiotic, cephalosporin, glycylcycline, penam, tetracycline antibiotic, rifamycin antibiotic, phenicol antibiotic, disinfecting agents and antiseptics	antibiotic target alteration, antibiotic efflux	28.79	73.72
Loose	ramA		protein homolog model	resistance-nodulation-cell division (RND) antibiotic efflux pump, General Bacterial Porin with reduced permeability to beta-lactams	fluoroquinolone antibiotic, monobactam, carbapenem, cephalosporin, glycylcycline, cephamycin, penam, tetracycline antibiotic, rifamycin antibiotic, phenicol antibiotic, penem, disinfecting agents and antiseptics	antibiotic efflux, reduced permeability to antibiotic	35.0	204.03
Loose	ramA		protein homolog model	resistance-nodulation-cell division (RND) antibiotic efflux pump, General Bacterial Porin with reduced permeability to beta-lactams	fluoroquinolone antibiotic, monobactam, carbapenem, cephalosporin, glycylcycline, cephamycin, penam, tetracycline antibiotic, rifamycin antibiotic, phenicol antibiotic, penem, disinfecting agents and antiseptics	antibiotic efflux, reduced permeability to antibiotic	29.41	293.55
Loose	RanA		protein homolog model	ATP-binding cassette (ABC) antibiotic efflux pump	aminoglycoside antibiotic	antibiotic efflux	36.13	128.68
Loose	RanA		protein homolog model	ATP-binding cassette (ABC) antibiotic efflux pump	aminoglycoside antibiotic	antibiotic efflux	31.69	139.15
Loose	RanA		protein homolog model	ATP-binding cassette (ABC) antibiotic efflux pump	aminoglycoside antibiotic	antibiotic efflux	31.3	97.29
Loose	RanA		protein homolog model	ATP-binding cassette (ABC) antibiotic efflux pump	aminoglycoside antibiotic	antibiotic efflux	31.25	96.51
Loose	RanA		protein homolog model	ATP-binding cassette (ABC) antibiotic efflux pump	aminoglycoside antibiotic	antibiotic efflux	30.98	153.88
Loose	RanA		protein homolog model	ATP-binding cassette (ABC) antibiotic efflux pump	aminoglycoside antibiotic	antibiotic efflux	30.42	141.09
Loose	RanA		protein homolog model	ATP-binding cassette (ABC) antibiotic efflux pump	aminoglycoside antibiotic	antibiotic efflux	30.17	142.25
Loose	RanA		protein homolog model	ATP-binding cassette (ABC) antibiotic efflux pump	aminoglycoside antibiotic	antibiotic efflux	29.84	127.13
Loose	RanA		protein homolog model	ATP-binding cassette (ABC) antibiotic efflux pump	aminoglycoside antibiotic	antibiotic efflux	29.8	143.41
Loose	RanA		protein homolog model	ATP-binding cassette (ABC) antibiotic efflux pump	aminoglycoside antibiotic	antibiotic efflux	29.65	127.91
Loose	RanA		protein homolog model	ATP-binding cassette (ABC) antibiotic efflux pump	aminoglycoside antibiotic	antibiotic efflux	29.29	138.37
Loose	RanA		protein homolog model	ATP-binding cassette (ABC) antibiotic efflux pump	aminoglycoside antibiotic	antibiotic efflux	29.22	102.71
Loose	patB		protein homolog model	ATP-binding cassette (ABC) antibiotic efflux pump	fluoroquinolone antibiotic	antibiotic efflux	31.05	109.52
Loose	patB		protein homolog model	ATP-binding cassette (ABC) antibiotic efflux pump	fluoroquinolone antibiotic	antibiotic efflux	30.19	98.64
Loose	patB		protein homolog model	ATP-binding cassette (ABC) antibiotic efflux pump0,8	fluoroquinolone antibiotic	antibiotic efflux	28.69	78.23
Loose	patB		protein homolog model	ATP-binding cassette (ABC) antibiotic efflux pump	fluoroquinolone antibiotic	antibiotic efflux	23.85	95.24
Loose	PDC-103		protein homolog model	PDC beta-lactamase	monobactam, carbapenem, cephalosporin	antibiotic inactivation	36.23	23.43
Loose	PmrF		protein homolog model	pmr phosphoethanolamine transferase	peptide antibiotic	antibiotic target alteration	24.85	109.94
Loose	POM-1		protein homolog model	POM beta-lactamase	carbapenem	antibiotic inactivation	38.3	79.37
Loose	poxtA		protein homolog model	Miscellaneous ABC-F subfamily ATP-binding cassette ribosomal protection proteins	tetracycline antibiotic, oxazolidinone antibiotic, phenicol antibiotic	antibiotic target protection	33.48	45.57
Loose	poxtA		protein homolog model	Miscellaneous ABC-F subfamily ATP-binding cassette ribosomal protection proteins	tetracycline antibiotic, oxazolidinone antibiotic, phenicol antibiotic	antibiotic target protection	24.92	72.14
Loose	Pseudomonas aeruginosa catB6		protein homolog model	chloramphenicol acetyltransferase (CAT)	phenicol antibiotic	antibiotic inactivation	60.91	99.05
Loose	Pseudomonas aeruginosa soxR		protein homolog model	ATP-binding cassette (ABC) antibiotic efflux pump, major facilitator superfamily (MFS) antibiotic efflux pump, resistance-nodulation-cell division (RND) antibiotic efflux pump	fluoroquinolone antibiotic, cephalosporin, glycylcycline, penam, tetracycline antibiotic, rifamycin antibiotic, phenicol antibiotic, disinfecting agents and antiseptics	antibiotic target alteration, antibiotic efflux	28.79	73.72
Loose	ramA		protein homolog model	resistance-nodulation-cell division (RND) antibiotic efflux pump, General Bacterial Porin with reduced permeability to beta-lactams	fluoroquinolone antibiotic, monobactam, carbapenem, cephalosporin, glycylcycline, cephamycin, penam, tetracycline antibiotic, rifamycin antibiotic, phenicol antibiotic, penem, disinfecting agents and antiseptics	antibiotic efflux, reduced permeability to antibiotic	35.0	204.03
Loose	ramA		protein homolog model	resistance-nodulation-cell division (RND) antibiotic efflux pump, General Bacterial Porin with reduced permeability to beta-lactams	fluoroquinolone antibiotic, monobactam, carbapenem, cephalosporin, glycylcycline, cephamycin, penam, tetracycline antibiotic, rifamycin antibiotic, phenicol antibiotic, penem, disinfecting agents and antiseptics	antibiotic efflux, reduced permeability to antibiotic	29.41	293.55
Loose	RanA		protein homolog model	ATP-binding cassette (ABC) antibiotic efflux pump	aminoglycoside antibiotic	antibiotic efflux	36.13	128.68
Loose	RanA		protein homolog model	ATP-binding cassette (ABC) antibiotic efflux pump	aminoglycoside antibiotic	antibiotic efflux	31.69	139.15
Loose	RanA		protein homolog model	ATP-binding cassette (ABC) antibiotic efflux pump	aminoglycoside antibiotic	antibiotic efflux	31.3	97.29
Loose	RanA		protein homolog model	ATP-binding cassette (ABC) antibiotic efflux pump	aminoglycoside antibiotic	antibiotic efflux	31.25	96.51

Loose	RanA		protein homolog model	ATP-binding cassette (ABC) antibiotic efflux pump	aminoglycoside antibiotic	antibiotic efflux	30.98	153.88
Loose	RanA		protein homolog model	ATP-binding cassette (ABC) antibiotic efflux pump	aminoglycoside antibiotic	antibiotic efflux	30.42	141.09
Loose	RanA		protein homolog model	ATP-binding cassette (ABC) antibiotic efflux pump	aminoglycoside antibiotic	antibiotic efflux	30.17	142.25
Loose	RanA		protein homolog model	ATP-binding cassette (ABC) antibiotic efflux pump	aminoglycoside antibiotic	antibiotic efflux	29.84	127.13
Loose	RanA		protein homolog model	ATP-binding cassette (ABC) antibiotic efflux pump	aminoglycoside antibiotic	antibiotic efflux	29.8	143.41
Loose	RanA		protein homolog model	ATP-binding cassette (ABC) antibiotic efflux pump	aminoglycoside antibiotic	antibiotic efflux	29.65	127.91
Loose	RanA		protein homolog model	ATP-binding cassette (ABC) antibiotic efflux pump	aminoglycoside antibiotic	antibiotic efflux	29.29	138.37
Loose	RanA		protein homolog model	ATP-binding cassette (ABC) antibiotic efflux pump	aminoglycoside antibiotic	antibiotic efflux	29.22	102.71
Loose	tetA(46)		protein homolog model	ATP-binding cassette (ABC) antibiotic efflux pump	tetracycline antibiotic	antibiotic efflux	60.0	11.32
Loose	tetA(46)		protein homolog model	ATP-binding cassette (ABC) antibiotic efflux pump	tetracycline antibiotic	antibiotic efflux	37.88	17.94
Loose	tetA(58)		protein homolog model	major facilitator superfamily (MFS) antibiotic efflux pump	tetracycline antibiotic	antibiotic efflux	41.15	70.85
Loose	tetA(58)		protein homolog model	major facilitator superfamily (MFS) antibiotic efflux pump	tetracycline antibiotic	antibiotic efflux	35.22	102.33
Loose	tetA(58)		protein homolog model	major facilitator superfamily (MFS) antibiotic efflux pump	tetracycline antibiotic	antibiotic efflux	35.2	87.76
Loose	tetA(58)		protein homolog model	major facilitator superfamily (MFS) antibiotic efflux pump	tetracycline antibiotic	antibiotic efflux	34.55	88.63
Loose	tetA(58)		protein homolog model	major facilitator superfamily (MFS) antibiotic efflux pump	tetracycline antibiotic	antibiotic efflux	34.21	72.89
Loose	tetA(58)		protein homolog model	major facilitator superfamily (MFS) antibiotic efflux pump	tetracycline antibiotic	antibiotic efflux	34.1	93.59
Loose	tetA(58)		protein homolog model	major facilitator superfamily (MFS) antibiotic efflux pump	tetracycline antibiotic	antibiotic efflux	34.08	72.89
Loose	tetA(58)		protein homolog model	major facilitator superfamily (MFS) antibiotic efflux pump	tetracycline antibiotic	antibiotic efflux	33.49	106.12
Loose	tetA(58)		protein homolog model	major facilitator superfamily (MFS) antibiotic efflux pump	tetracycline antibiotic	antibiotic efflux	30.5	78.72
Loose	tetA(58)		protein homolog model	major facilitator superfamily (MFS) antibiotic efflux pump	tetracycline antibiotic	antibiotic efflux	27.35	67.93
Loose	tetA(58)		protein homolog model	major facilitator superfamily (MFS) antibiotic efflux pump	tetracycline antibiotic	antibiotic efflux	27.11	72.01
Loose	tetA(58)		protein homolog model	major facilitator superfamily (MFS) antibiotic efflux pump	tetracycline antibiotic	antibiotic efflux	26.85	67.06
Loose	tetA(58)		protein homolog model	major facilitator superfamily (MFS) antibiotic efflux pump	tetracycline antibiotic	antibiotic efflux	25.57	149.27
Loose	tetA(58)		protein homolog model	major facilitator superfamily (MFS) antibiotic efflux pump	tetracycline antibiotic	antibiotic efflux	24.78	78.13
Loose	tetA(60)		protein homolog model	ATP-binding cassette (ABC) antibiotic efflux pump	tetracycline antibiotic	antibiotic efflux	31.65	99.83
Loose	tetB(60)		protein homolog model	ATP-binding cassette (ABC) antibiotic efflux pump	tetracycline antibiotic	antibiotic efflux	23.64	92.57
Loose	tetM		protein homolog model	tetracycline-resistant ribosomal protection protein	tetracycline antibiotic	antibiotic target protection	31.94	108.92
Loose	tetT		protein homolog model	tetracycline-resistant ribosomal protection protein	tetracycline antibiotic	antibiotic target protection	28.74	106.76
Loose	tsnR		protein homolog model	non-erm 23S ribosomal RNA methyltransferase (A1067)	peptide antibiotic	antibiotic target alteration	26.85	88.52
Loose	TxR		protein homolog model	ATP-binding cassette (ABC) antibiotic efflux pump	tetracycline antibiotic	antibiotic efflux	37.99	146.86
Loose	TxR		protein homolog model	ATP-binding cassette (ABC) antibiotic efflux pump	tetracycline antibiotic	antibiotic efflux	28.2	171.38
Loose	ugd		protein homolog model	pmr phosphoethanolamine transferase	peptide antibiotic	antibiotic target alteration	71.73	86.60
Loose	vanH gene in vanA cluster		protein homolog model	vanH, glycopeptide resistance gene cluster	glycopeptide antibiotic	antibiotic target alteration	33.06	99.07
Loose	vanH gene in vanA cluster		protein homolog model	vanH, glycopeptide resistance gene cluster	glycopeptide antibiotic	antibiotic target alteration	30.94	97.52
Loose	vanH gene in vanA cluster		protein homolog model	vanH, glycopeptide resistance gene cluster	glycopeptide antibiotic	antibiotic target alteration	24.23	101.55
Loose	vanH gene in vanF cluster		protein homolog model	vanH, glycopeptide resistance gene cluster	glycopeptide antibiotic	antibiotic target alteration	34.06	103.42
Loose	vanL		protein homolog model	glycopeptide resistance gene cluster, Van ligase	glycopeptide antibiotic	antibiotic target alteration	25.3	84.24
Loose	vanN		protein homolog model	glycopeptide resistance gene cluster, Van ligase	glycopeptide antibiotic	antibiotic target alteration	34.25	229.74
Loose	vanR gene in vanB cluster		protein homolog model	glycopeptide resistance gene cluster, vanR	glycopeptide antibiotic	antibiotic target alteration	31.03	54.55
Loose	vanR gene in vanB cluster		protein homolog model	glycopeptide resistance gene cluster, vanR	glycopeptide antibiotic	antibiotic target alteration	30.08	112.73
Loose	vanR gene in vanE cluster		protein homolog model	glycopeptide resistance gene cluster, vanR	glycopeptide antibiotic	antibiotic target alteration	35.78	51.09
Loose	vanR gene in vanE cluster		protein homolog model	glycopeptide resistance gene cluster, vanR	glycopeptide antibiotic	antibiotic target alteration	34.78	96.94
Loose	vanR gene in vanE cluster		protein homolog model	glycopeptide resistance gene cluster, vanR	glycopeptide antibiotic	antibiotic target alteration	33.86	167.25

Loose	vanR gene in vanE cluster		protein homolog model	glycopeptide resistance gene cluster, vanR	glycopeptide antibiotic	antibiotic target alteration	29.41	283.84
Loose	vanR gene in vanF cluster		protein homolog model	glycopeptide resistance gene cluster, vanR	glycopeptide antibiotic	antibiotic target alteration	43.86	96.97
Loose	vanR gene in vanF cluster		protein homolog model	glycopeptide resistance gene cluster, vanR	glycopeptide antibiotic	antibiotic target alteration	36.73	96.10
Loose	vanR gene in vanG cluster		protein homolog model	glycopeptide resistance gene cluster, vanR	glycopeptide antibiotic	antibiotic target alteration	34.11	57.02
Loose	vanR gene in vanI cluster		protein homolog model	glycopeptide resistance gene cluster, vanR	glycopeptide antibiotic	antibiotic target alteration	40.0	51.29
Loose	vanR gene in vanI cluster		protein homolog model	glycopeptide resistance gene cluster, vanR	glycopeptide antibiotic	antibiotic target alteration	25.0	55.17
Loose	vanS gene in vanL cluster		protein homolog model	vanS, glycopeptide resistance gene cluster	glycopeptide antibiotic	antibiotic target alteration	29.74	250.00
Loose	vanS gene in vanL cluster		protein homolog model	vanS, glycopeptide resistance gene cluster	glycopeptide antibiotic	antibiotic target alteration	29.44	138.46
Loose	vanS gene in vanL cluster		protein homolog model	vanS, glycopeptide resistance gene cluster	glycopeptide antibiotic	antibiotic target alteration	25.17	112.91
Loose	vanS gene in vanM cluster		protein homolog model	vanS, glycopeptide resistance gene cluster	glycopeptide antibiotic	antibiotic target alteration	23.74	125.41
Loose	vanT gene in vanG cluster		protein homolog model	glycopeptide resistance gene cluster, vanT	glycopeptide antibiotic	antibiotic target alteration	33.07	52.25
Loose	vanU gene in vanG cluster		protein homolog model	glycopeptide resistance gene cluster, vanU	glycopeptide antibiotic	antibiotic target alteration	50.0	74.67
Loose	vatC		protein homolog model	streptogramin vat acetyltransferase	streptogramin antibiotic, streptogramin A antibiotic	antibiotic inactivation	32.11	141.04
Loose	vatD		protein homolog model	streptogramin vat acetyltransferase	streptogramin antibiotic, streptogramin A antibiotic	antibiotic inactivation	34.51	90.69
Loose	vatH		protein homolog model	streptogramin vat acetyltransferase	streptogramin antibiotic, streptogramin A antibiotic	antibiotic inactivation	35.9	106.94
Loose	vatH		protein homolog model	streptogramin vat acetyltransferase	streptogramin antibiotic, streptogramin A antibiotic	antibiotic inactivation	25.69	206.94
Loose	vgaB		protein homolog model	vga-type ABC-F protein	streptogramin antibiotic, streptogramin A antibiotic, pleuromutilin antibiotic	antibiotic target protection	47.62	77.36
Loose	YajC		protein homolog model	resistance-nodulation-cell division (RND) antibiotic efflux pump	A fluoroquinolone antibiotic, cephalosporin, glycylcycline, penam, a tetracycline antibiotic, rifamycin antibiotic, phenicol antibiotic, disinfecting agents, and antiseptics	antibiotic efflux	35.64	142.86
Loose	YEM-1		protein homolog model	YEM beta-lactamase	carbapenem	antibiotic inactivation	23.97	115.04

RGI Criteria	ARO Term	SNP	Detection Criteria	AMR Gene Family	Drug Class	Resistance Mechanism	% Identity of Matching Region	% Length od Refence Sequence
Loose	AAC(6')-Isa		protein homolog model	AAC(6')	aminoglycoside antibiotic	antibiotic inactivation	25.14	115.29
Loose	aadK		protein homolog model	ANT(6)	aminoglycoside antibiotic	antibiotic inactivation	37.94	102.11
Loose	AcrS		protein homolog model	resistance-nodulation-cell division (RND) antibiotic efflux pump	fluoroquinolone antibiotic, cephalosporin, glycylcycline, cephamycin, penam, tetracycline antibiotic, rifamycin antibiotic, phenicol antibiotic, disinfecting agents and antiseptics	antibiotic efflux	21.26	90.00
Loose	adeL		protein homolog model	resistance-nodulation-cell division (RND) antibiotic efflux pump	fluoroquinolone antibiotic, tetracycline antibiotic	antibiotic efflux	23.01	86.65
Loose	adeN		protein homolog model	resistance-nodulation-cell division (RND) antibiotic efflux pump	macrolide antibiotic, fluoroquinolone antibiotic, lincosamide antibiotic, carbapenem, cephalosporin, tetracycline antibiotic, rifamycin antibiotic, diaminopyrimidine antibiotic, phenicol antibiotic, penem	antibiotic efflux	31.36	91.71
Loose	adeR		protein homolog model	resistance-nodulation-cell division (RND) antibiotic efflux pump	glycylcycline, tetracycline antibiotic	antibiotic efflux	34.58	140.08
Loose	adeR		protein homolog model	resistance-nodulation-cell division (RND) antibiotic efflux pump	glycylcycline, tetracycline antibiotic	antibiotic efflux	24.44	95.14
Loose	Agrobacterium fabrum chloramphenicol acetyltransferase		protein homolog model	chloramphenicol acetyltransferase (CAT)	phenicol antibiotic	antibiotic inactivation	43.14	107.18
Loose	APH(2'')-IIIa		protein homolog model	APH(2'')	aminoglycoside antibiotic	antibiotic inactivation	25.23	89.22
Loose	apmA		protein homolog model	amp acetyltransferase	aminoglycoside antibiotic	antibiotic inactivation	39.44	73.36
Loose	arlR		protein homolog model	major facilitator superfamily (MFS) antibiotic efflux pump	fluoroquinolone antibiotic, disinfecting agents and antiseptics	antibiotic efflux	47.06	101.37
Loose	arlR		protein homolog model	major facilitator superfamily (MFS) antibiotic efflux pump	fluoroquinolone antibiotic, disinfecting agents and antiseptics	antibiotic efflux	33.18	102.28
Loose	arlR		protein homolog model	major facilitator superfamily (MFS) antibiotic efflux pump	fluoroquinolone antibiotic, disinfecting agents and antiseptics	antibiotic efflux	30.0	53.88
Loose	arlS		protein homolog model	major facilitator superfamily (MFS) antibiotic efflux pump	fluoroquinolone antibiotic, disinfecting agents and antiseptics	antibiotic efflux	34.04	95.12
Loose	arlS		protein homolog model	major facilitator superfamily (MFS) antibiotic efflux pump	fluoroquinolone antibiotic, disinfecting agents and antiseptics	antibiotic efflux	30.21	40.80
Loose	arlS		protein homolog model	major facilitator superfamily (MFS) antibiotic efflux pump	fluoroquinolone antibiotic, disinfecting agents and antiseptics	antibiotic efflux	28.89	70.29
Loose	arlS		protein homolog model	major facilitator superfamily (MFS) antibiotic efflux pump	fluoroquinolone antibiotic, disinfecting agents and antiseptics	antibiotic efflux	28.7	98.89
Loose	arlS		protein homolog model	major facilitator superfamily (MFS) antibiotic efflux pump	fluoroquinolone antibiotic, disinfecting agents and antiseptics	antibiotic efflux	27.31	229.49
Loose	arlS		protein homolog model	major facilitator superfamily (MFS) antibiotic efflux pump	fluoroquinolone antibiotic, disinfecting agents and antiseptics	antibiotic efflux	26.73	74.94
Loose	arlS		protein homolog model	major facilitator superfamily (MFS) antibiotic efflux pump	fluoroquinolone antibiotic, disinfecting agents and antiseptics	antibiotic efflux	25.81	104.21
Loose	arlS		protein homolog model	major facilitator superfamily (MFS) antibiotic efflux pump	fluoroquinolone antibiotic, disinfecting agents and antiseptics	antibiotic efflux	24.55	98.67
Loose	arnA		protein homolog model	pmr phosphoethanolamine transferase	peptide antibiotic	antibiotic target alteration	33.33	48.79
Loose	arnA		protein homolog model	pmr phosphoethanolamine transferase	peptide antibiotic	antibiotic target alteration	25.95	49.09
Loose	arnA		protein homolog model	pmr phosphoethanolamine transferase	peptide antibiotic	antibiotic target alteration	25.6	47.13
Loose	bacA		protein homolog model	undecaprenyl pyrophosphate related proteins	peptide antibiotic	antibiotic target alteration	28.79	92.67
Loose	BcII		protein homolog model	Bc beta-lactamase	cephalosporin, penam	antibiotic inactivation	25.32	73.05
Loose	bcrA		protein homolog model	ATP-binding cassette (ABC) antibiotic efflux pump	peptide antibiotic	antibiotic efflux	38.43	81.37
Loose	bcrA		protein homolog model	ATP-binding cassette (ABC) antibiotic efflux pump	peptide antibiotic	antibiotic efflux	38.43	81.37
Loose	bcrA		protein homolog model	ATP-binding cassette (ABC) antibiotic efflux pump	peptide antibiotic	antibiotic efflux	36.13	93.46
Loose	bcrA		protein homolog model	ATP-binding cassette (ABC) antibiotic efflux pump	peptide antibiotic	antibiotic efflux	34.5	82.35
Loose	bcrA		protein homolog model	ATP-binding cassette (ABC) antibiotic efflux pump	peptide antibiotic	antibiotic efflux	34.39	98.04

Loose	bcrA		protein homolog model	ATP-binding cassette (ABC) antibiotic efflux pump	peptide antibiotic	antibiotic efflux	34.12	96.73
Loose	bcrA		protein homolog model	ATP-binding cassette (ABC) antibiotic efflux pump	peptide antibiotic	antibiotic efflux	33.33	172.22
Loose	bcrA		protein homolog model	ATP-binding cassette (ABC) antibiotic efflux pump	peptide antibiotic	antibiotic efflux	31.78	77.78
Loose	bcrA		protein homolog model	ATP-binding cassette (ABC) antibiotic efflux pump	peptide antibiotic	antibiotic efflux	31.28	76.14
Loose	bcrA		protein homolog model	ATP-binding cassette (ABC) antibiotic efflux pump	peptide antibiotic	antibiotic efflux	31.12	100.33
Loose	bcrA		protein homolog model	ATP-binding cassette (ABC) antibiotic efflux pump	peptide antibiotic	antibiotic efflux	31.08	92.81
Loose	bcrA		protein homolog model	ATP-binding cassette (ABC) antibiotic efflux pump	peptide antibiotic	antibiotic efflux	30.87	76.80
Loose	bcrA		protein homolog model	ATP-binding cassette (ABC) antibiotic efflux pump	peptide antibiotic	antibiotic efflux	30.65	87.91
Loose	bcrA		protein homolog model	ATP-binding cassette (ABC) antibiotic efflux pump	peptide antibiotic	antibiotic efflux	30.09	85.62
Loose	bcrA		protein homolog model	ATP-binding cassette (ABC) antibiotic efflux pump	peptide antibiotic	antibiotic efflux	28.57	83.01
Loose	bcrA		protein homolog model	ATP-binding cassette (ABC) antibiotic efflux pump	peptide antibiotic	antibiotic efflux	26.34	97.71
Loose	bcrA		protein homolog model	ATP-binding cassette (ABC) antibiotic efflux pump	peptide antibiotic	antibiotic efflux	26.27	162.75
Loose	bcrA		protein homolog model	ATP-binding cassette (ABC) antibiotic efflux pump	peptide antibiotic	antibiotic efflux	25.67	92.48
Loose	Borreliella burgdorferi murA with mutation conferring resistance to fosfomycin	D116C	protein variant model	antibiotic-resistant murA transferase	Fosfomycin	antibiotic target alteration	35.61	100.70
Loose	catB8		protein homolog model	chloramphenicol acetyltransferase (CAT)	phenicol antibiotic	antibiotic inactivation	47.69	89.52
Loose	cdeA		protein homolog model	multidrug and toxic compound extrusion (MATE) transporter	fluoroquinolone antibiotic, disinfecting agents and antiseptics	antibiotic efflux	40.32	102.49
Loose	cdeA		protein homolog model	multidrug and toxic compound extrusion (MATE) transporter	fluoroquinolone antibiotic, disinfecting agents and antiseptics	antibiotic efflux	35.38	99.55
Loose	cdeA		protein homolog model	multidrug and toxic compound extrusion (MATE) transporter	fluoroquinolone antibiotic, disinfecting agents and antiseptics	antibiotic efflux	34.19	100.45
Loose	cdeA		protein homolog model	multidrug and toxic compound extrusion (MATE) transporter	fluoroquinolone antibiotic, disinfecting agents and antiseptics	antibiotic efflux	31.28	100.00
Loose	cdeA		protein homolog model	multidrug and toxic compound extrusion (MATE) transporter	fluoroquinolone antibiotic, disinfecting agents and antiseptics	antibiotic efflux	30.64	100.45
Loose	cdeA		protein homolog model	multidrug and toxic compound extrusion (MATE) transporter	fluoroquinolone antibiotic, disinfecting agents and antiseptics	antibiotic efflux	26.32	106.58
Loose	cdeA		protein homolog model	multidrug and toxic compound extrusion (MATE) transporter	fluoroquinolone antibiotic, disinfecting agents and antiseptics	antibiotic efflux	26.21	99.32
Loose	cdeA		protein homolog model	multidrug and toxic compound extrusion (MATE) transporter	fluoroquinolone antibiotic, disinfecting agents and antiseptics	antibiotic efflux	26.03	102.04
Loose	cdeA		protein homolog model	multidrug and toxic compound extrusion (MATE) transporter	fluoroquinolone antibiotic, disinfecting agents and antiseptics	antibiotic efflux	23.47	101.36
Loose	cfr(D)		protein homolog model	Cfr 23S ribosomal RNA methyltransferase	lincosamide antibiotic, streptogramin antibiotic, oxazolidinone antibiotic, phenicol antibiotic	antibiotic target alteration	18.4	86.27
Loose	clbA		protein homolog model	Cfr 23S ribosomal RNA methyltransferase	lincosamide antibiotic, streptogramin antibiotic, streptogramin A antibiotic, oxazolidinone antibiotic, phenicol antibiotic, pleuromutilin antibiotic	antibiotic target alteration	34.95	97.99
Loose	Clostridioides difficile gyrA conferring resistance to fluoroquinolones	P116A	protein variant model	fluoroquinolone resistant gyrA	fluoroquinolone antibiotic	antibiotic target alteration	44.65	102.10
Loose	Clostridioides difficile gyrB conferring	V423F	protein variant model	fluoroquinolone resistant gyrB	fluoroquinolone antibiotic	antibiotic target alteration	51.79	100.63

	resistance to fluoroquinolones							
Loose	cmeA		protein homolog model	resistance-nodulation-cell division (RND) antibiotic efflux pump	macrolide antibiotic, fluoroquinolone antibiotic, cephalosporin, fusidic acid	antibiotic efflux	25.11	88.28
Loose	cmeR		protein homolog model	resistance-nodulation-cell division (RND) antibiotic efflux pump	macrolide antibiotic, fluoroquinolone antibiotic, cephalosporin, fusidic acid	antibiotic efflux	39.29	97.14
Loose	cmeR		protein homolog model	resistance-nodulation-cell division (RND) antibiotic efflux pump	macrolide antibiotic, fluoroquinolone antibiotic, cephalosporin, fusidic acid	antibiotic efflux	27.19	98.10
Loose	cpxA		protein homolog model	resistance-nodulation-cell division (RND) antibiotic efflux pump	aminoglycoside antibiotic, aminocoumarin antibiotic	antibiotic efflux	28.43	29.32
Loose	cpxA		protein homolog model	resistance-nodulation-cell division (RND) antibiotic efflux pump	aminoglycoside antibiotic, aminocoumarin antibiotic	antibiotic efflux	26.09	86.65
Loose	CRD3-1		protein homolog model	CRD3 beta-lactamase	carbapenem	antibiotic inactivation	41.27	77.56
Loose	CRP		protein homolog model	resistance-nodulation-cell division (RND) antibiotic efflux pump	macrolide antibiotic, fluoroquinolone antibiotic, penam	antibiotic efflux	22.33	110.48
Loose	EdeQ		protein homolog model	Edeine acetyltransferase	peptide antibiotic, polyamine antibiotic	antibiotic inactivation	38.13	97.24
Loose	efrA		protein homolog model	ATP-binding cassette (ABC) antibiotic efflux pump	macrolide antibiotic, fluoroquinolone antibiotic, rifamycin antibiotic	antibiotic efflux	46.86	128.00
Loose	Erm(48)		protein homolog model	Erm 23S ribosomal RNA methyltransferase	macrolide antibiotic, lincosamide antibiotic, streptogramin antibiotic	antibiotic target alteration	28.77	109.05
Loose	ErmD		protein homolog model	Erm 23S ribosomal RNA methyltransferase	macrolide antibiotic, lincosamide antibiotic, streptogramin antibiotic, streptogramin A antibiotic, streptogramin B antibiotic	antibiotic target alteration	25.53	87.46
Loose	ErmE		protein homolog model	Erm 23S ribosomal RNA methyltransferase	macrolide antibiotic, lincosamide antibiotic, streptogramin antibiotic, streptogramin A antibiotic, streptogramin B antibiotic	antibiotic target alteration	26.07	69.03
Loose	Escherichia coli AcrAB-TolC with AcrR mutation conferring resistance to ciprofloxacin, tetracycline, and ceftazidime		protein overexpression model	resistance-nodulation-cell division (RND) antibiotic efflux pump	fluoroquinolone antibiotic, cephalosporin, glycylcycline, penam, tetracycline antibiotic, rifamycin antibiotic, phenicol antibiotic, disinfecting agents and antiseptics	antibiotic target alteration, antibiotic efflux	38.24	101.40
Loose	Escherichia coli AcrAB-TolC with AcrR mutation conferring resistance to ciprofloxacin, tetracycline, and ceftazidime		protein overexpression model	resistance-nodulation-cell division (RND) antibiotic efflux pump	fluoroquinolone antibiotic, cephalosporin, glycylcycline, penam, tetracycline antibiotic, rifamycin antibiotic, phenicol antibiotic, disinfecting agents and antiseptics	antibiotic target alteration, antibiotic efflux	30.0	174.42
Loose	Escherichia coli AcrAB-TolC with AcrR mutation conferring resistance to ciprofloxacin, tetracycline, and ceftazidime		protein overexpression model	resistance-nodulation-cell division (RND) antibiotic efflux pump	fluoroquinolone antibiotic, cephalosporin, glycylcycline, penam, tetracycline antibiotic, rifamycin antibiotic, phenicol antibiotic, disinfecting agents and antiseptics	antibiotic target alteration, antibiotic efflux	30.0	89.30
Loose	Escherichia coli ampC1 beta-lactamase		protein homolog model	ampC-type beta-lactamase	cephalosporin, penam	antibiotic inactivation	36.71	123.04
Loose	Escherichia coli ampC1 beta-lactamase		protein homolog model	ampC-type beta-lactamase	cephalosporin, penam	antibiotic inactivation	26.32	76.73
Loose	Escherichia coli EF-Tu mutants conferring	R234F	protein variant model	elfamycin resistant EF-Tu	elfamycin antibiotic	antibiotic target alteration	68.34	97.56

	resistance to Pulvomycin							
Loose	Escherichia coli PtsI with mutation conferring resistance to fosfomycin	V25I	protein variant model	antibiotic-resistant ptsI phosphotransferase	fosfomycin	antibiotic target alteration	43.72	98.26
Loose	Escherichia coli soxS with mutation conferring antibiotic resistance		protein overexpression model	ATP-binding cassette (ABC) antibiotic efflux pump, major facilitator superfamily (MFS) antibiotic efflux pump, resistance-nodulation-cell division (RND) antibiotic efflux pump, General Bacterial Porin with reduced permeability to beta-lactams	fluoroquinolone antibiotic, monobactam, carbapenem, cephalosporin, glycylcycline, cephamycin, penam, tetracycline antibiotic, rifamycin antibiotic, phenicol antibiotic, penem, disinfecting agents and antiseptics	antibiotic target alteration, antibiotic efflux, reduced permeability to antibiotic	31.63	295.33
Loose	Escherichia coli soxS with mutation conferring antibiotic resistance		protein overexpression model	ATP-binding cassette (ABC) antibiotic efflux pump, major facilitator superfamily (MFS) antibiotic efflux pump, resistance-nodulation-cell division (RND) antibiotic efflux pump, General Bacterial Porin with reduced permeability to beta-lactams	fluoroquinolone antibiotic, monobactam, carbapenem, cephalosporin, glycylcycline, cephamycin, penam, tetracycline antibiotic, rifamycin antibiotic, phenicol antibiotic, penem, disinfecting agents and antiseptics	antibiotic target alteration, antibiotic efflux, reduced permeability to antibiotic	27.72	235.51
Loose	Escherichia coli soxS with mutation conferring antibiotic resistance		protein overexpression model	ATP-binding cassette (ABC) antibiotic efflux pump, major facilitator superfamily (MFS) antibiotic efflux pump, resistance-nodulation-cell division (RND) antibiotic efflux pump, General Bacterial Porin with reduced permeability to beta-lactams	fluoroquinolone antibiotic, monobactam, carbapenem, cephalosporin, glycylcycline, cephamycin, penam, tetracycline antibiotic, rifamycin antibiotic, phenicol antibiotic, penem, disinfecting agents and antiseptics	antibiotic target alteration, antibiotic efflux, reduced permeability to antibiotic	25.74	265.42
Loose	evgA		protein homolog model	major facilitator superfamily (MFS) antibiotic efflux pump, resistance-nodulation-cell division (RND) antibiotic efflux pump	macrolide antibiotic, fluoroquinolone antibiotic, penam, tetracycline antibiotic	antibiotic efflux	26.17	107.35
Loose	evgA		protein homolog model	major facilitator superfamily (MFS) antibiotic efflux pump, resistance-nodulation-cell division (RND) antibiotic efflux pump	macrolide antibiotic, fluoroquinolone antibiotic, penam, tetracycline antibiotic	antibiotic efflux	23.91	93.63
Loose	evgS		protein homolog model	major facilitator superfamily (MFS) antibiotic efflux pump, resistance-nodulation-cell division (RND) antibiotic efflux pump	macrolide antibiotic, fluoroquinolone antibiotic, penam, tetracycline antibiotic	antibiotic efflux	29.92	53.80
Loose	evgS		protein homolog model	major facilitator superfamily (MFS) antibiotic efflux pump, resistance-nodulation-cell division (RND) antibiotic efflux pump	macrolide antibiotic, fluoroquinolone antibiotic, penam, tetracycline antibiotic	antibiotic efflux	28.84	74.52
Loose	evgS		protein homolog model	major facilitator superfamily (MFS) antibiotic efflux pump, resistance-nodulation-cell division (RND) antibiotic efflux pump	macrolide antibiotic, fluoroquinolone antibiotic, penam, tetracycline antibiotic	antibiotic efflux	27.08	41.77
Loose	evgS		protein homolog model	major facilitator superfamily (MFS) antibiotic efflux pump, resistance-nodulation-cell division (RND) antibiotic efflux pump	macrolide antibiotic, fluoroquinolone antibiotic, penam, tetracycline antibiotic	antibiotic efflux	26.24	55.56
Loose	GOB-46		protein homolog model	GOB beta-lactamase	carbapenem, cephalosporin, penam	antibiotic inactivation	27.67	88.62
Loose	golS		protein homolog model	resistance-nodulation-cell division (RND) antibiotic efflux pump	monobactam, carbapenem, cephalosporin, cephamycin, penam, phenicol antibiotic, penem	antibiotic efflux	28.97	184.42
Loose	golS		protein homolog model	resistance-nodulation-cell division (RND) antibiotic efflux pump	monobactam, carbapenem, cephalosporin, cephamycin, penam, phenicol antibiotic, penem	antibiotic efflux	24.27	95.45
Loose	golS		protein homolog model	resistance-nodulation-cell division (RND) antibiotic efflux pump	monobactam, carbapenem, cephalosporin, cephamycin, penam, phenicol antibiotic, penem	antibiotic efflux	23.85	161.69
Loose	Helicobacter pylori pbp1 mutants conferring resistance to amoxicillin	S414N, T593S	protein variant model	Penicillin-binding protein mutations conferring resistance to beta-lactam antibiotics	cephalosporin, cephamycin, penam	antibiotic target alteration	28.69	105.77
Loose	hmrM		protein homolog model	multidrug and toxic compound extrusion (MATE) transporter	fluoroquinolone antibiotic, disinfecting agents and antiseptics	antibiotic efflux	27.36	97.41
Loose	hp1165		protein homolog model	major facilitator superfamily (MFS) antibiotic efflux pump	tetracycline antibiotic	antibiotic efflux	24.8	101.55
Loose	IMP-25		protein homolog model	IMP beta-lactamase	carbapenem, cephalosporin, cephamycin, penam, penem	antibiotic inactivation	25.15	97.97

Loose	kdpE		protein homolog model	kdpDE	aminoglycoside antibiotic	antibiotic efflux	36.11	154.67
Loose	kdpE		protein homolog model	kdpDE	aminoglycoside antibiotic	antibiotic efflux	25.23	60.89
Loose	LlmA 23S ribosomal RNA methyltransferase		protein homolog model	Llm 23S ribosomal RNA methyltransferase	lincosamide antibiotic	antibiotic target alteration	28.91	134.15
Loose	lmrD		protein homolog model	ATP-binding cassette (ABC) antibiotic efflux pump	lincosamide antibiotic	antibiotic efflux	31.93	80.87
Loose	LpsB		protein homolog model	Intrinsic peptide antibiotic resistant Lps	peptide antibiotic	reduced permeability to antibiotic	24.22	104.64
Loose	lsaC		protein homolog model	lsa-type ABC-F protein	lincosamide antibiotic, streptogramin antibiotic, pleuromutilin antibiotic	antibiotic target protection	38.08	104.47
Loose	lsaC		protein homolog model	lsa-type ABC-F protein	lincosamide antibiotic, streptogramin antibiotic, pleuromutilin antibiotic	antibiotic target protection	31.91	52.24
Loose	lsaC		protein homolog model	lsa-type ABC-F protein	lincosamide antibiotic, streptogramin antibiotic, pleuromutilin antibiotic	antibiotic target protection	26.46	49.39
Loose	lsaC		protein homolog model	lsa-type ABC-F protein	lincosamide antibiotic, streptogramin antibiotic, pleuromutilin antibiotic	antibiotic target protection	23.43	115.04
Loose	macB		protein homolog model	ATP-binding cassette (ABC) antibiotic efflux pump	macrolide antibiotic	antibiotic efflux	46.93	35.87
Loose	macB		protein homolog model	ATP-binding cassette (ABC) antibiotic efflux pump	macrolide antibiotic	antibiotic efflux	38.84	35.56
Loose	macB		protein homolog model	ATP-binding cassette (ABC) antibiotic efflux pump	macrolide antibiotic	antibiotic efflux	38.03	37.42
Loose	macB		protein homolog model	ATP-binding cassette (ABC) antibiotic efflux pump	macrolide antibiotic	antibiotic efflux	37.56	34.94
Loose	macB		protein homolog model	ATP-binding cassette (ABC) antibiotic efflux pump	macrolide antibiotic	antibiotic efflux	37.39	39.44
Loose	macB		protein homolog model	ATP-binding cassette (ABC) antibiotic efflux pump	macrolide antibiotic	antibiotic efflux	34.4	63.51
Loose	macB		protein homolog model	ATP-binding cassette (ABC) antibiotic efflux pump	macrolide antibiotic	antibiotic efflux	34.38	38.82
Loose	macB		protein homolog model	ATP-binding cassette (ABC) antibiotic efflux pump	macrolide antibiotic	antibiotic efflux	32.98	145.81
Loose	macB		protein homolog model	ATP-binding cassette (ABC) antibiotic efflux pump	macrolide antibiotic	antibiotic efflux	31.87	50.00
Loose	macB		protein homolog model	ATP-binding cassette (ABC) antibiotic efflux pump	macrolide antibiotic	antibiotic efflux	30.58	48.91
Loose	macB		protein homolog model	ATP-binding cassette (ABC) antibiotic efflux pump	macrolide antibiotic	antibiotic efflux	30.51	49.53
Loose	macB		protein homolog model	ATP-binding cassette (ABC) antibiotic efflux pump	macrolide antibiotic	antibiotic efflux	30.42	54.04
Loose	macB		protein homolog model	ATP-binding cassette (ABC) antibiotic efflux pump	macrolide antibiotic	antibiotic efflux	29.86	40.99
Loose	macB		protein homolog model	ATP-binding cassette (ABC) antibiotic efflux pump	macrolide antibiotic	antibiotic efflux	29.22	31.68
Loose	macB		protein homolog model	ATP-binding cassette (ABC) antibiotic efflux pump	macrolide antibiotic	antibiotic efflux	29.13	77.33
Loose	macB		protein homolog model	ATP-binding cassette (ABC) antibiotic efflux pump	macrolide antibiotic	antibiotic efflux	27.47	50.62
Loose	macB		protein homolog model	ATP-binding cassette (ABC) antibiotic efflux pump	macrolide antibiotic	antibiotic efflux	27.08	178.26
Loose	macB		protein homolog model	ATP-binding cassette (ABC) antibiotic efflux pump	macrolide antibiotic	antibiotic efflux	26.89	51.09
Loose	macB		protein homolog model	ATP-binding cassette (ABC) antibiotic efflux pump	macrolide antibiotic	antibiotic efflux	25.5	52.64
Loose	marA		protein homolog model	resistance-nodulation-cell division (RND) antibiotic efflux pump, General Bacterial Porin with reduced permeability to beta-lactams	fluoroquinolone antibiotic, monobactam, carbapenem, cephalosporin, glycylcycline, cephamycin, penam, tetracycline antibiotic, rifamycin antibiotic, phenicol antibiotic, penem, disinfecting agents and antiseptics	antibiotic efflux, reduced permeability to antibiotic	37.5	221.26
Loose	marA		protein homolog model	resistance-nodulation-cell division (RND) antibiotic efflux pump, General Bacterial Porin with reduced permeability to beta-lactams	fluoroquinolone antibiotic, monobactam, carbapenem, cephalosporin, glycylcycline, cephamycin, penam, tetracycline antibiotic, rifamycin antibiotic, phenicol antibiotic, penem, disinfecting agents and antiseptics	antibiotic efflux, reduced permeability to antibiotic	29.13	255.91
Loose	marA		protein homolog model	resistance-nodulation-cell division (RND) antibiotic efflux pump, General Bacterial Porin with reduced permeability to beta-lactams	fluoroquinolone antibiotic, monobactam, carbapenem, cephalosporin, glycylcycline, cephamycin, penam, tetracycline antibiotic, rifamycin antibiotic, phenicol antibiotic, penem, disinfecting agents and antiseptics	antibiotic efflux, reduced permeability to antibiotic	26.32	251.18
Loose	mdtC		protein homolog model	resistance-nodulation-cell division (RND) antibiotic efflux pump	aminocoumarin antibiotic	antibiotic efflux	25.33	88.20
Loose	MdtK		protein homolog model	multidrug and toxic compound extrusion (MATE) transporter	fluoroquinolone antibiotic	antibiotic efflux	20.91	93.67
Loose	mecA		protein homolog model	methicillin resistant PBP2	penam	antibiotic target replacement	26.25	85.03
Loose	mefH		protein homolog model	major facilitator superfamily (MFS) antibiotic efflux pump	macrolide antibiotic	antibiotic efflux	39.25	369.72
Loose	mel		protein homolog model	msr-type ABC-F protein	macrolide antibiotic, streptogramin antibiotic	antibiotic target protection	23.82	103.21
Loose	mepA		protein homolog model	multidrug and toxic compound extrusion (MATE) transporter	glycylcycline, tetracycline antibiotic	antibiotic efflux	31.78	101.11

Loose	mepA		protein homolog model	multidrug and toxic compound extrusion (MATE) transporter	glycylcycline, tetracycline antibiotic	antibiotic efflux	27.68	100.00
Loose	mepA		protein homolog model	multidrug and toxic compound extrusion (MATE) transporter	glycylcycline, tetracycline antibiotic	antibiotic efflux	24.94	100.44
Loose	mepA		protein homolog model	multidrug and toxic compound extrusion (MATE) transporter	glycylcycline, tetracycline antibiotic	antibiotic efflux	23.74	101.33
Loose	mepA		protein homolog model	multidrug and toxic compound extrusion (MATE) transporter	glycylcycline, tetracycline antibiotic	antibiotic efflux	21.63	100.22
Loose	mepR		protein homolog model	multidrug and toxic compound extrusion (MATE) transporter	glycylcycline, tetracycline antibiotic	antibiotic efflux	23.01	105.76
Loose	mgrA		protein homolog model	ATP-binding cassette (ABC) antibiotic efflux pump, major facilitator superfamily (MFS) antibiotic efflux pump	fluoroquinolone antibiotic, cephalosporin, penam, tetracycline antibiotic, peptide antibiotic, disinfecting agents and antiseptics	antibiotic efflux	31.69	95.92
Loose	mgrA		protein homolog model	ATP-binding cassette (ABC) antibiotic efflux pump, major facilitator superfamily (MFS) antibiotic efflux pump	fluoroquinolone antibiotic, cephalosporin, penam, tetracycline antibiotic, peptide antibiotic, disinfecting agents and antiseptics	antibiotic efflux	29.03	99.32
Loose	mgrA		protein homolog model	ATP-binding cassette (ABC) antibiotic efflux pump, major facilitator superfamily (MFS) antibiotic efflux pump	fluoroquinolone antibiotic, cephalosporin, penam, tetracycline antibiotic, peptide antibiotic, disinfecting agents and antiseptics	antibiotic efflux	28.57	102.04
Loose	mgrA		protein homolog model	ATP-binding cassette (ABC) antibiotic efflux pump, major facilitator superfamily (MFS) antibiotic efflux pump	fluoroquinolone antibiotic, cephalosporin, penam, tetracycline antibiotic, peptide antibiotic, disinfecting agents and antiseptics	antibiotic efflux	26.23	100.68
Loose	mgrA		protein homolog model	ATP-binding cassette (ABC) antibiotic efflux pump, major facilitator superfamily (MFS) antibiotic efflux pump	fluoroquinolone antibiotic, cephalosporin, penam, tetracycline antibiotic, peptide antibiotic, disinfecting agents and antiseptics	antibiotic efflux	24.8	102.72
Loose	mreA		protein homolog model	major facilitator superfamily (MFS) antibiotic efflux pump	macrolide antibiotic	antibiotic efflux	31.06	94.86
Loose	msbA		protein homolog model	ATP-binding cassette (ABC) antibiotic efflux pump	nitroimidazole antibiotic	antibiotic efflux	32.09	99.48
Loose	msbA		protein homolog model	ATP-binding cassette (ABC) antibiotic efflux pump	nitroimidazole antibiotic	antibiotic efflux	30.84	98.63
Loose	msbA		protein homolog model	ATP-binding cassette (ABC) antibiotic efflux pump	nitroimidazole antibiotic	antibiotic efflux	30.04	101.03
Loose	msbA		protein homolog model	ATP-binding cassette (ABC) antibiotic efflux pump	nitroimidazole antibiotic	antibiotic efflux	29.37	100.52
Loose	msbA		protein homolog model	ATP-binding cassette (ABC) antibiotic efflux pump	nitroimidazole antibiotic	antibiotic efflux	29.18	102.41
Loose	msbA		protein homolog model	ATP-binding cassette (ABC) antibiotic efflux pump	nitroimidazole antibiotic	antibiotic efflux	29.06	100.34
Loose	msbA		protein homolog model	ATP-binding cassette (ABC) antibiotic efflux pump	nitroimidazole antibiotic	antibiotic efflux	28.63	45.19
Loose	msbA		protein homolog model	ATP-binding cassette (ABC) antibiotic efflux pump	nitroimidazole antibiotic	antibiotic efflux	28.57	55.67
Loose	msbA		protein homolog model	ATP-binding cassette (ABC) antibiotic efflux pump	nitroimidazole antibiotic	antibiotic efflux	27.52	44.50
Loose	msbA		protein homolog model	ATP-binding cassette (ABC) antibiotic efflux pump	nitroimidazole antibiotic	antibiotic efflux	27.27	44.16
Loose	msbA		protein homolog model	ATP-binding cassette (ABC) antibiotic efflux pump	nitroimidazole antibiotic	antibiotic efflux	21.34	42.96
Loose	mtrA		protein homolog model	resistance-nodulation-cell division (RND) antibiotic efflux pump	macrolide antibiotic, penam	antibiotic efflux	44.87	106.14
Loose	mtrA		protein homolog model	resistance-nodulation-cell division (RND) antibiotic efflux pump	macrolide antibiotic, penam	antibiotic efflux	36.53	96.49
Loose	mtrA		protein homolog model	resistance-nodulation-cell division (RND) antibiotic efflux pump	macrolide antibiotic, penam	antibiotic efflux	29.66	53.07
Loose	Mycobacterium tuberculosis kasA mutant conferring resistance to isoniazid	D66N	protein variant model	antibiotic resistant kasA	isoniazid	antibiotic target alteration	32.76	98.80
Loose	Mycobacterium tuberculosis pncA mutations conferring resistance to pyrazinamide	T47S, W119R	protein variant model	Pyrazinamide resistant pncA	Pyrazinamide]	antibiotic target alteration	28.49	104.30
Loose	Mycobacterium tuberculosis pncA mutations conferring	D136N, H59D, G97S	protein variant model	Pyrazinamide resistant pncA	pyrazinamide	antibiotic target alteration	25.68	101.08

	resistance to pyrazinamide							
Loose	Mycobacterium tuberculosis rpoB mutants conferring resistance to rifampicin	H526S, I572F, D516G, S512R, T508A	protein variant model	rifamycin-resistant beta-subunit of RNA polymerase (rpoB)	rifamycin antibiotic	antibiotic target alteration, antibiotic target replacement	48.11	101.28
Loose	Mycolicibacterium smegmatis ndh with mutation conferring resistance to isoniazid	G84D, Q335H	protein variant model	antibiotic resistant ndh	isoniazid	antibiotic target alteration	31.55	137.20
Loose	nalD		protein overexpression model	resistance-nodulation-cell division (RND) antibiotic efflux pump	macrolide antibiotic, fluoroquinolone antibiotic, monobactam, carbapenem, cephalosporin, cephamycin, penam, tetracycline antibiotic, peptide antibiotic, aminocoumarin antibiotic, diaminopyrimidine antibiotic, sulfonamide antibiotic, phenicol antibiotic, penem	antibiotic efflux	30.26	114.15
Loose	nalD		protein overexpression model	resistance-nodulation-cell division (RND) antibiotic efflux pump	macrolide antibiotic, fluoroquinolone antibiotic, monobactam, carbapenem, cephalosporin, cephamycin, penam, tetracycline antibiotic, peptide antibiotic, aminocoumarin antibiotic, diaminopyrimidine antibiotic, sulfonamide antibiotic, phenicol antibiotic, penem	antibiotic efflux	25.42	175.94
Loose	norB		protein homolog model	major facilitator superfamily (MFS) antibiotic efflux pump	fluoroquinolone antibiotic	antibiotic efflux	24.86	85.19
Loose	novA		protein homolog model	ATP-binding cassette (ABC) antibiotic efflux pump	aminocoumarin antibiotic	antibiotic efflux	40.87	97.24
Loose	novA		protein homolog model	ATP-binding cassette (ABC) antibiotic efflux pump	aminocoumarin antibiotic	antibiotic efflux	36.04	63.58
Loose	novA		protein homolog model	ATP-binding cassette (ABC) antibiotic efflux pump	aminocoumarin antibiotic	antibiotic efflux	32.47	93.50
Loose	optrA		protein homolog model	Miscellaneous ABC-F subfamily ATP-binding cassette ribosomal protection proteins	oxazolidinone antibiotic, phenicol antibiotic	antibiotic target protection	29.59	91.45
Loose	optrA		protein homolog model	Miscellaneous ABC-F subfamily ATP-binding cassette ribosomal protection proteins	oxazolidinone antibiotic, phenicol antibiotic	antibiotic target protection	24.16	71.76
Loose	patA		protein homolog model	ATP-binding cassette (ABC) antibiotic efflux pump	fluoroquinolone antibiotic	antibiotic efflux	28.15	45.04
Loose	patA		protein homolog model	ATP-binding cassette (ABC) antibiotic efflux pump	fluoroquinolone antibiotic	antibiotic efflux	27.95	43.97
Loose	patA		protein homolog model	ATP-binding cassette (ABC) antibiotic efflux pump	fluoroquinolone antibiotic	antibiotic efflux	27.52	43.97
Loose	patB		protein homolog model	ATP-binding cassette (ABC) antibiotic efflux pump	fluoroquinolone antibiotic	antibiotic efflux	35.94	38.61
Loose	patB		protein homolog model	ATP-binding cassette (ABC) antibiotic efflux pump	fluoroquinolone antibiotic	antibiotic efflux	34.69	104.93
Loose	patB		protein homolog model	ATP-binding cassette (ABC) antibiotic efflux pump	fluoroquinolone antibiotic	antibiotic efflux	31.05	109.52
Loose	patB		protein homolog model	ATP-binding cassette (ABC) antibiotic efflux pump	fluoroquinolone antibiotic	antibiotic efflux	30.19	98.64
Loose	patB		protein homolog model	ATP-binding cassette (ABC) antibiotic efflux pump	fluoroquinolone antibiotic	antibiotic efflux	28.69	78.23
Loose	patB		protein homolog model	ATP-binding cassette (ABC) antibiotic efflux pump	fluoroquinolone antibiotic	antibiotic efflux	23.85	95.24
Loose	PDC-103		protein homolog model	PDC beta-lactamase	monobactam, carbapenem, cephalosporin	antibiotic inactivation	36.23	23.43
Loose	PmrF		protein homolog model	pmr phosphoethanolamine transferase	peptide antibiotic	antibiotic target alteration	24.85	109.94
Loose	POM-1		protein homolog model	POM beta-lactamase	carbapenem	antibiotic inactivation	38.3	79.37
Loose	poxtA		protein homolog model	Miscellaneous ABC-F subfamily ATP-binding cassette ribosomal protection proteins	tetracycline antibiotic, oxazolidinone antibiotic, phenicol antibiotic	antibiotic target protection	33.48	45.57
Loose	poxtA		protein homolog model	Miscellaneous ABC-F subfamily ATP-binding cassette ribosomal protection proteins	tetracycline antibiotic, oxazolidinone antibiotic, phenicol antibiotic	antibiotic target protection	24.92	72.14
Loose	Pseudomonas aeruginosa catB6		protein homolog model	chloramphenicol acetyltransferase (CAT)	phenicol antibiotic	antibiotic inactivation	60.91	99.05
Loose	Pseudomonas aeruginosa soxR		protein homolog model	ATP-binding cassette (ABC) antibiotic efflux pump, major facilitator superfamily (MFS) antibiotic efflux pump, resistance-nodulation-cell division (RND) antibiotic efflux pump	fluoroquinolone antibiotic, cephalosporin, glycylcycline, penam, tetracycline antibiotic, rifamycin antibiotic, phenicol antibiotic, disinfecting agents and antiseptics	antibiotic target alteration, antibiotic efflux	28.79	73.72
Loose	ramA		protein homolog model	resistance-nodulation-cell division (RND) antibiotic efflux pump, General Bacterial Porin with reduced permeability to beta-lactams	fluoroquinolone antibiotic, monobactam, carbapenem, cephalosporin, glycylcycline, cephamycin, penam, tetracycline antibiotic, rifamycin antibiotic, phenicol antibiotic, penem, disinfecting agents and antiseptics	antibiotic efflux, reduced permeability to antibiotic	35.0	204.03

Loose	ramA		protein homolog model	resistance-nodulation-cell division (RND) antibiotic efflux pump, General Bacterial Porin with reduced permeability to beta-lactams	fluoroquinolone antibiotic, monobactam, carbapenem, cephalosporin, glycylcycline, cephamycin, penam, tetracycline antibiotic, rifamycin antibiotic, phenicol antibiotic, penem, disinfecting agents and antiseptics	antibiotic efflux, reduced permeability to antibiotic	29.41	293.55
Loose	RanA		protein homolog model	ATP-binding cassette (ABC) antibiotic efflux pump	aminoglycoside antibiotic	antibiotic efflux	36.13	128.68
Loose	RanA		protein homolog model	ATP-binding cassette (ABC) antibiotic efflux pump	aminoglycoside antibiotic	antibiotic efflux	31.69	139.15
Loose	RanA		protein homolog model	ATP-binding cassette (ABC) antibiotic efflux pump	aminoglycoside antibiotic	antibiotic efflux	31.3	97.29
Loose	RanA		protein homolog model	ATP-binding cassette (ABC) antibiotic efflux pump	aminoglycoside antibiotic	antibiotic efflux	31.25	96.51
Loose	RanA		protein homolog model	ATP-binding cassette (ABC) antibiotic efflux pump	aminoglycoside antibiotic	antibiotic efflux	30.98	153.88
Loose	RanA		protein homolog model	ATP-binding cassette (ABC) antibiotic efflux pump	aminoglycoside antibiotic	antibiotic efflux	30.42	141.09
Loose	RanA		protein homolog model	ATP-binding cassette (ABC) antibiotic efflux pump	aminoglycoside antibiotic	antibiotic efflux	30.17	142.25
Loose	RanA		protein homolog model	ATP-binding cassette (ABC) antibiotic efflux pump	aminoglycoside antibiotic	antibiotic efflux	29.84	127.13
Loose	RanA		protein homolog model	ATP-binding cassette (ABC) antibiotic efflux pump	aminoglycoside antibiotic	antibiotic efflux	29.8	143.41
Loose	RanA		protein homolog model	ATP-binding cassette (ABC) antibiotic efflux pump	aminoglycoside antibiotic	antibiotic efflux	29.65	127.91
Loose	RanA		protein homolog model	ATP-binding cassette (ABC) antibiotic efflux pump	aminoglycoside antibiotic	antibiotic efflux	29.29	138.37
Loose	RanA		protein homolog model	ATP-binding cassette (ABC) antibiotic efflux pump	aminoglycoside antibiotic	antibiotic efflux	29.22	102.71
Loose	patB		protein homolog model	ATP-binding cassette (ABC) antibiotic efflux pump	fluoroquinolone antibiotic	antibiotic efflux	31.05	109.52
Loose	patB		protein homolog model	ATP-binding cassette (ABC) antibiotic efflux pump	fluoroquinolone antibiotic	antibiotic efflux	30.19	98.64
Loose	patB		protein homolog model	ATP-binding cassette (ABC) antibiotic efflux pump0,8	fluoroquinolone antibiotic	antibiotic efflux	28.69	78.23
Loose	patB		protein homolog model	ATP-binding cassette (ABC) antibiotic efflux pump	fluoroquinolone antibiotic	antibiotic efflux	23.85	95.24
Loose	PDC-103		protein homolog model	PDC beta-lactamase	monobactam, carbapenem, cephalosporin	antibiotic inactivation	36.23	23.43
Loose	PmrF		protein homolog model	pmr phosphoethanolamine transferase	peptide antibiotic	antibiotic target alteration	24.85	109.94
Loose	POM-1		protein homolog model	POM beta-lactamase	carbapenem	antibiotic inactivation	38.3	79.37
Loose	poxtA		protein homolog model	Miscellaneous ABC-F subfamily ATP-binding cassette ribosomal protection proteins	tetracycline antibiotic, oxazolidinone antibiotic, phenicol antibiotic	antibiotic target protection	33.48	45.57
Loose	poxtA		protein homolog model	Miscellaneous ABC-F subfamily ATP-binding cassette ribosomal protection proteins	tetracycline antibiotic, oxazolidinone antibiotic, phenicol antibiotic	antibiotic target protection	24.92	72.14
Loose	Pseudomonas aeruginosa catB6		protein homolog model	chloramphenicol acetyltransferase (CAT)	phenicol antibiotic	antibiotic inactivation	60.91	99.05
Loose	Pseudomonas aeruginosa soxR		protein homolog model	ATP-binding cassette (ABC) antibiotic efflux pump, major facilitator superfamily (MFS) antibiotic efflux pump, resistance-nodulation-cell division (RND) antibiotic efflux pump	fluoroquinolone antibiotic, cephalosporin, glycylcycline, penam, tetracycline antibiotic, rifamycin antibiotic, phenicol antibiotic, disinfecting agents and antiseptics	antibiotic target alteration, antibiotic efflux	28.79	73.72
Loose	ramA		protein homolog model	resistance-nodulation-cell division (RND) antibiotic efflux pump, General Bacterial Porin with reduced permeability to beta-lactams	fluoroquinolone antibiotic, monobactam, carbapenem, cephalosporin, glycylcycline, cephamycin, penam, tetracycline antibiotic, rifamycin antibiotic, phenicol antibiotic, penem, disinfecting agents and antiseptics	antibiotic efflux, reduced permeability to antibiotic	35.0	204.03
Loose	ramA		protein homolog model	resistance-nodulation-cell division (RND) antibiotic efflux pump, General Bacterial Porin with reduced permeability to beta-lactams	fluoroquinolone antibiotic, monobactam, carbapenem, cephalosporin, glycylcycline, cephamycin, penam, tetracycline antibiotic, rifamycin antibiotic, phenicol antibiotic, penem, disinfecting agents and antiseptics	antibiotic efflux, reduced permeability to antibiotic	29.41	293.55
Loose	RanA		protein homolog model	ATP-binding cassette (ABC) antibiotic efflux pump	aminoglycoside antibiotic	antibiotic efflux	36.13	128.68
Loose	RanA		protein homolog model	ATP-binding cassette (ABC) antibiotic efflux pump	aminoglycoside antibiotic	antibiotic efflux	31.69	139.15
Loose	RanA		protein homolog model	ATP-binding cassette (ABC) antibiotic efflux pump	aminoglycoside antibiotic	antibiotic efflux	31.3	97.29
Loose	RanA		protein homolog model	ATP-binding cassette (ABC) antibiotic efflux pump	aminoglycoside antibiotic	antibiotic efflux	31.25	96.51
Loose	RanA		protein homolog model	ATP-binding cassette (ABC) antibiotic efflux pump	aminoglycoside antibiotic	antibiotic efflux	30.98	153.88
Loose	RanA		protein homolog model	ATP-binding cassette (ABC) antibiotic efflux pump	aminoglycoside antibiotic	antibiotic efflux	30.42	141.09
Loose	RanA		protein homolog model	ATP-binding cassette (ABC) antibiotic efflux pump	aminoglycoside antibiotic	antibiotic efflux	30.17	142.25
Loose	RanA		protein homolog model	ATP-binding cassette (ABC) antibiotic efflux pump	aminoglycoside antibiotic	antibiotic efflux	29.84	127.13
Loose	RanA		protein homolog model	ATP-binding cassette (ABC) antibiotic efflux pump	aminoglycoside antibiotic	antibiotic efflux	29.8	143.41
Loose	RanA		protein homolog model	ATP-binding cassette (ABC) antibiotic efflux pump	aminoglycoside antibiotic	antibiotic efflux	29.65	127.91
Loose	RanA		protein homolog model	ATP-binding cassette (ABC) antibiotic efflux pump	aminoglycoside antibiotic	antibiotic efflux	29.29	138.37
Loose	RanA		protein homolog model	ATP-binding cassette (ABC) antibiotic efflux pump	aminoglycoside antibiotic	antibiotic efflux	29.22	102.71
Loose	tetA(46)		protein homolog model	ATP-binding cassette (ABC) antibiotic efflux pump	tetracycline antibiotic	antibiotic efflux	60.0	11.32
Loose	tetA(46)		protein homolog model	ATP-binding cassette (ABC) antibiotic efflux pump	tetracycline antibiotic	antibiotic efflux	37.88	17.94
Loose	tetA(58)		protein homolog model	major facilitator superfamily (MFS) antibiotic efflux pump	tetracycline antibiotic	antibiotic efflux	41.15	70.85
Loose	tetA(58)		protein homolog model	major facilitator superfamily (MFS) antibiotic efflux pump	tetracycline antibiotic	antibiotic efflux	35.22	102.33

Loose	tetA(58)		protein homolog model	major facilitator superfamily (MFS) antibiotic efflux pump	tetracycline antibiotic	antibiotic efflux	35.2	87.76
Loose	tetA(58)		protein homolog model	major facilitator superfamily (MFS) antibiotic efflux pump	tetracycline antibiotic	antibiotic efflux	34.55	88.63
Loose	tetA(58)		protein homolog model	major facilitator superfamily (MFS) antibiotic efflux pump	tetracycline antibiotic	antibiotic efflux	34.21	72.89
Loose	tetA(58)		protein homolog model	major facilitator superfamily (MFS) antibiotic efflux pump	tetracycline antibiotic	antibiotic efflux	34.1	93.59
Loose	tetA(58)		protein homolog model	major facilitator superfamily (MFS) antibiotic efflux pump	tetracycline antibiotic	antibiotic efflux	34.08	72.89
Loose	tetA(58)		protein homolog model	major facilitator superfamily (MFS) antibiotic efflux pump	tetracycline antibiotic	antibiotic efflux	33.49	106.12
Loose	tetA(58)		protein homolog model	major facilitator superfamily (MFS) antibiotic efflux pump	tetracycline antibiotic	antibiotic efflux	30.5	78.72
Loose	tetA(58)		protein homolog model	major facilitator superfamily (MFS) antibiotic efflux pump	tetracycline antibiotic	antibiotic efflux	27.35	67.93
Loose	tetA(58)		protein homolog model	major facilitator superfamily (MFS) antibiotic efflux pump	tetracycline antibiotic	antibiotic efflux	27.11	72.01
Loose	tetA(58)		protein homolog model	major facilitator superfamily (MFS) antibiotic efflux pump	tetracycline antibiotic	antibiotic efflux	26.85	67.06
Loose	tetA(58)		protein homolog model	major facilitator superfamily (MFS) antibiotic efflux pump	tetracycline antibiotic	antibiotic efflux	25.57	149.27
Loose	tetA(58)		protein homolog model	major facilitator superfamily (MFS) antibiotic efflux pump	tetracycline antibiotic	antibiotic efflux	24.78	78.13
Loose	tetA(60)		protein homolog model	ATP-binding cassette (ABC) antibiotic efflux pump	tetracycline antibiotic	antibiotic efflux	31.65	99.83
Loose	tetB(60)		protein homolog model	ATP-binding cassette (ABC) antibiotic efflux pump	tetracycline antibiotic	antibiotic efflux	23.64	92.57
Loose	tetM		protein homolog model	tetracycline-resistant ribosomal protection protein	tetracycline antibiotic	antibiotic target protection	31.94	108.92
Loose	tetT		protein homolog model	tetracycline-resistant ribosomal protection protein	tetracycline antibiotic	antibiotic target protection	28.74	106.76
Loose	tsnR		protein homolog model	non-erm 23S ribosomal RNA methyltransferase (A1067)	peptide antibiotic	antibiotic target alteration	26.85	88.52
Loose	TxR		protein homolog model	ATP-binding cassette (ABC) antibiotic efflux pump	tetracycline antibiotic	antibiotic efflux	37.99	146.86
Loose	TxR		protein homolog model	ATP-binding cassette (ABC) antibiotic efflux pump	tetracycline antibiotic	antibiotic efflux	28.2	171.38
Loose	ugd		protein homolog model	pmr phosphoethanolamine transferase	peptide antibiotic	antibiotic target alteration	71.73	86.60
Loose	vanH gene in vanA cluster		protein homolog model	vanH, glycopeptide resistance gene cluster	glycopeptide antibiotic	antibiotic target alteration	33.06	99.07
Loose	vanH gene in vanA cluster		protein homolog model	vanH, glycopeptide resistance gene cluster	glycopeptide antibiotic	antibiotic target alteration	30.94	97.52
Loose	vanH gene in vanA cluster		protein homolog model	vanH, glycopeptide resistance gene cluster	glycopeptide antibiotic	antibiotic target alteration	24.23	101.55
Loose	vanH gene in vanF cluster		protein homolog model	vanH, glycopeptide resistance gene cluster	glycopeptide antibiotic	antibiotic target alteration	34.06	103.42
Loose	vanL		protein homolog model	glycopeptide resistance gene cluster, Van ligase	glycopeptide antibiotic	antibiotic target alteration	25.3	84.24
Loose	vanN		protein homolog model	glycopeptide resistance gene cluster, Van ligase	glycopeptide antibiotic	antibiotic target alteration	34.25	229.74
Loose	vanR gene in vanB cluster		protein homolog model	glycopeptide resistance gene cluster, vanR	glycopeptide antibiotic	antibiotic target alteration	31.03	54.55
Loose	vanR gene in vanB cluster		protein homolog model	glycopeptide resistance gene cluster, vanR	glycopeptide antibiotic	antibiotic target alteration	30.08	112.73
Loose	vanR gene in vanE cluster		protein homolog model	glycopeptide resistance gene cluster, vanR	glycopeptide antibiotic	antibiotic target alteration	35.78	51.09
Loose	vanR gene in vanE cluster		protein homolog model	glycopeptide resistance gene cluster, vanR	glycopeptide antibiotic	antibiotic target alteration	34.78	96.94
Loose	vanR gene in vanE cluster		protein homolog model	glycopeptide resistance gene cluster, vanR	glycopeptide antibiotic	antibiotic target alteration	33.86	167.25
Loose	vanR gene in vanE cluster		protein homolog model	glycopeptide resistance gene cluster, vanR	glycopeptide antibiotic	antibiotic target alteration	29.41	283.84
Loose	vanR gene in vanF cluster		protein homolog model	glycopeptide resistance gene cluster, vanR	glycopeptide antibiotic	antibiotic target alteration	43.86	96.97
Loose	vanR gene in vanF cluster		protein homolog model	glycopeptide resistance gene cluster, vanR	glycopeptide antibiotic	antibiotic target alteration	36.73	96.10
Loose	vanR gene in vanG cluster		protein homolog model	glycopeptide resistance gene cluster, vanR	glycopeptide antibiotic	antibiotic target alteration	34.11	57.02
Loose	vanR gene in vanI cluster		protein homolog model	glycopeptide resistance gene cluster, vanR	glycopeptide antibiotic	antibiotic target alteration	40.0	51.29
Loose	vanR gene in vanI cluster		protein homolog model	glycopeptide resistance gene cluster, vanR	glycopeptide antibiotic	antibiotic target alteration	25.0	55.17

Loose	vanS gene in vanL cluster		protein homolog model	vanS, glycopeptide resistance gene cluster	glycopeptide antibiotic	antibiotic target alteration	29.74	250.00
Loose	vanS gene in vanL cluster		protein homolog model	vanS, glycopeptide resistance gene cluster	glycopeptide antibiotic	antibiotic target alteration	29.44	138.46
Loose	vanS gene in vanL cluster		protein homolog model	vanS, glycopeptide resistance gene cluster	glycopeptide antibiotic	antibiotic target alteration	25.17	112.91
Loose	vanS gene in vanM cluster		protein homolog model	vanS, glycopeptide resistance gene cluster	glycopeptide antibiotic	antibiotic target alteration	23.74	125.41
Loose	vanT gene in vanG cluster		protein homolog model	glycopeptide resistance gene cluster, vanT	glycopeptide antibiotic	antibiotic target alteration	33.07	52.25
Loose	vanU gene in vanG cluster		protein homolog model	glycopeptide resistance gene cluster, vanU	glycopeptide antibiotic	antibiotic target alteration	50.0	74.67
Loose	vatC		protein homolog model	streptogramin vat acetyltransferase	streptogramin antibiotic, streptogramin A antibiotic	antibiotic inactivation	32.11	141.04
Loose	vatD		protein homolog model	streptogramin vat acetyltransferase	streptogramin antibiotic, streptogramin A antibiotic	antibiotic inactivation	34.51	90.69
Loose	vatH		protein homolog model	streptogramin vat acetyltransferase	streptogramin antibiotic, streptogramin A antibiotic	antibiotic inactivation	35.9	106.94
Loose	vatH		protein homolog model	streptogramin vat acetyltransferase	streptogramin antibiotic, streptogramin A antibiotic	antibiotic inactivation	25.69	206.94
Loose	vgaB		protein homolog model	vga-type ABC-F protein	streptogramin antibiotic, streptogramin A antibiotic, pleuromutilin antibiotic	antibiotic target protection	47.62	77.36
Loose	YajC		protein homolog model	resistance-nodulation-cell division (RND) antibiotic efflux pump	A fluoroquinolone antibiotic, cephalosporin, glycylcycline, penam, a tetracycline antibiotic, rifamycin antibiotic, phenicol antibiotic, disinfecting agents, and antiseptics	antibiotic efflux	35.64	142.86
Loose	YEM-1		protein homolog model	YEM beta-lactamase	carbapenem	antibiotic inactivation	23.97	115.04
